# *Candidatus* Methylumidiphilus Drives Peaks in Methanotrophic Relative Abundance in Stratified Lakes and Ponds Across Northern Landscapes

**DOI:** 10.3389/fmicb.2021.669937

**Published:** 2021-08-12

**Authors:** Gaëtan Martin, Antti J. Rissanen, Sarahi L. Garcia, Maliheh Mehrshad, Moritz Buck, Sari Peura

**Affiliations:** ^1^Department of Forest Mycology and Plant Pathology, Science for Life Laboratory, Swedish University of Agricultural Sciences, Uppsala, Sweden; ^2^Faculty of Engineering and Natural Sciences, Tampere University, Tampere, Finland; ^3^Department of Ecology, Environment and Plant Sciences, Science for Life Laboratory, Stockholm University, Stockholm, Sweden; ^4^Department of Aquatic Sciences and Assessment, Swedish University of Agricultural Sciences, Uppsala, Sweden

**Keywords:** methanotroph, greenhouse gas, metagenomics, thaw ponds, microbial diversity, lakes

## Abstract

Boreal lakes and ponds produce two-thirds of the total natural methane emissions above the latitude of 50° North. These lake emissions are regulated by methanotrophs which can oxidize up to 99% of the methane produced in the sediments and the water column. Despite their importance, the diversity and distribution of the methanotrophs in lakes are still poorly understood. Here, we used shotgun metagenomic data to explore the diversity and distribution of methanotrophs in 40 oxygen-stratified water bodies in boreal and subarctic areas in Europe and North America. In our data, gammaproteobacterial methanotrophs (order *Methylococcales*) generally dominated the methanotrophic communities throughout the water columns. A recently discovered lineage of *Methylococcales*, *Candidatus* Methylumidiphilus, was present in all the studied water bodies and dominated the methanotrophic community in lakes with a high relative abundance of methanotrophs. Alphaproteobacterial methanotrophs were the second most abundant group of methanotrophs. In the top layer of the lakes, characterized by low CH_4_ concentration, their abundance could surpass that of the gammaproteobacterial methanotrophs. These results support the theory that the alphaproteobacterial methanotrophs have a high affinity for CH_4_ and can be considered stress-tolerant strategists. In contrast, the gammaproteobacterial methanotrophs are competitive strategists. In addition, relative abundances of anaerobic methanotrophs, *Candidatus* Methanoperedenaceae and *Candidatus* Methylomirabilis, were strongly correlated, suggesting possible co-metabolism. Our data also suggest that these anaerobic methanotrophs could be active even in the oxic layers. In non-metric multidimensional scaling, alpha- and gammaproteobacterial methanotrophs formed separate clusters based on their abundances in the samples, except for the gammaproteobacterial *Candidatus* Methylumidiphilus, which was separated from these two clusters. This may reflect similarities in the niche and environmental requirements of the different genera within alpha- and gammaproteobacterial methanotrophs. Our study confirms the importance of O_2_ and CH_4_ in shaping the methanotrophic communities and suggests that one variable cannot explain the diversity and distribution of the methanotrophs across lakes. Instead, we suggest that the diversity and distribution of freshwater methanotrophs are regulated by lake-specific factors.

## Introduction

Whereas anthropogenic carbon dioxide (CO_2_) has been the most important greenhouse gas (GHG) since the early days of the industrial era, most recent estimates indicate that methane (CH_4_) has been responsible for a quarter of cumulative radiative forcing for CO_2_, CH_4_, and nitrous oxide (Etminan et al., 2016). Several studies suggest that lakes and ponds are the dominant and underestimated sources of natural CH_4_ emissions at high northern latitudes (in boreal and arctic areas) (Bastviken et al., 2011; Wik et al., 2016a, b). Furthermore, the physics and biology of lakes are all expected to change globally due to direct human activities and climate change, which might lead to increased CH_4_ emissions (Tranvik et al., 2009; Wik et al., 2016b). For this reason, it is of utmost importance to gather more information on the organisms and processes behind the CH_4_ emissions.

CH_4_ emissions from lakes are a net balance between methane production by methanogens and consumption by methane oxidizers [methanotrophs (MO)]. According to the estimates, MO can consume between 30 and 99% of CH_4_ produced in the sediments and the water column before it reaches the atmosphere (Frenzel et al., 1990; Kankaala et al., 2006; Bastviken et al., 2008; Mayr et al., 2020b). The extent of emissions depends on the efficiency of the methanotrophic biofilter and environmental conditions, such as mixing patterns, ebullition, and trophic state of the lakes (Kankaala et al., 2007; Bellido et al., 2009; Yang et al., 2019). Increased temperature and eutrophication are expected to surge the CH_4_ production in lakes (Sepulveda-Jauregui et al., 2018; Zhou et al., 2020). However, those could also improve the efficiency of the methanotrophic biofilter (Davidson et al., 2015; Denfeld et al., 2018; de Jong et al., 2018). A better knowledge of the diversity and distribution of methanotrophic communities is essential for understanding the biological mechanisms behind the dynamic methane equilibrium and eventually predicting possible future changes in the functioning of the CH_4_ biofilter (Wagg et al., 2019).

**FIGURE 1:**
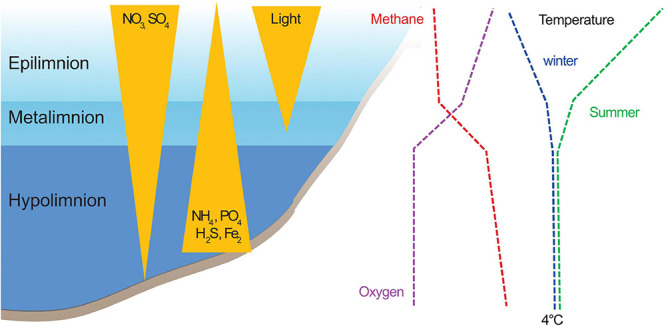
Schematic representation of a stratified lake. During stratification, lake water column is divided into three distinct layers. Whereas the stability and depth of these layers depend on several parameters, such as temperature, salinity, color, morphology of the lake, climate, etc.; the different layers tend to share a few common features. The surface layer, *epilimnion*, is rich in oxygen while at the lake bottom, in the hypolimnion, anaerobic conditions prevail. Between these two layers, a thin but distinct layer develops, where temperature and oxygen change drastically, this is the *metalimnion*. The condition in the hypolimnion are favorable for reduced compounds, such as CH_4_, NH_4_, or H_2_S, whereas in the epilimnion, conditions favor oxidation. Concentration curves of reduced and oxidized compounds often cross each other in the metalimnion (e.g., oxygen and methane). During winter, when the lake freezes, the surface water gets colder (i.e., below 4°C) and the temperatures of the layers are inverted, with the warmer layer being the hypolimnion. The depths of each of the layers are not the same during winter and summer stratification.

Oxygen-stratified lakes are hotspots for CH_4_ oxidizing bacteria. Known methanotrophs inhabit and are active throughout the water column but are typically most abundant in the metalimnion (Sundh et al., 2005; Pimenov et al., 2010; Samad and Bertilsson, 2017; Rissanen et al., 2018; Reis et al., 2020). The metalimnion is characterized by decreasing oxygen and temperature and increasing nutrient and CH_4_ concentrations (Figure 1). Thus, layers low in oxygen and high in CH_4_ are considered hubs for CH_4_ oxidation (Bastviken et al., 2004; Pimenov et al., 2010; Blees et al., 2014). In those conditions, as well as in oxygen saturated water, CH_4_ oxidation is considered to be performed mainly by aerobic methanotrophs belonging to alpha- and gammaproteobacteria (Taipale et al., 2011; Tsutsumi et al., 2011; Blees et al., 2014; Biderre-Petit et al., 2019; Reis et al., 2020). Methane oxidizers of the recently discovered acidophilic genus *Methylacidiphilum* in the phylum Verrucomicrobia (V-MO) (Camp et al., 2009) are also using O_2_ as an electron acceptor but are associated with extreme environments (Sharp et al., 2014; van Teeseling et al., 2014; Schmitz et al., 2021). The taxa involved in CH_4_ oxidation in anoxic environments include Archaea (ANME archaea, referred to as MOA in the following text) (Valentine, 2002) and Bacteria belonging to genus *Candidatus* Methylomirabilis (in the phylum NC10, referred to as NC10-MO in the following text) (Raghoebarsing et al., 2006). These taxa use alternative electron acceptors, such as SO_4_^2–^ and NO_3_^–^NO_2_^–^ instead of O_2_ (Valentine, 2002; Beal et al., 2009; Wu et al., 2011; Oswald et al., 2017). Furthermore, recent studies suggest that some gammaproteobacterial methane oxidizers (ƴ-MO) have the potential for fermentation (Kalyuzhnaya et al., 2013; Gilman et al., 2017) and anaerobic respiration (Kits et al., 2015; Oswald et al., 2016; Zheng et al., 2020). The importance of MO as a methane biofilter in anoxic freshwaters is still unclear (Reed et al., 2017). However, it is known that anaerobic MO can consume large quantities of CH_4_ and represent a substantial portion of the microbial biomass in the anoxic layer of the lakes (hypolimnion) when conditions are favorable (Graf et al., 2018).

Some recent studies suggest that the O_2_ and CH_4_ counter gradients are responsible for niche partitioning of alphaproteobacterial methane oxidizers (α-MO) and ƴ-MO and underline how this partitioning might be essential for predicting the efficiency of the CH_4_ biofilter (Mayr et al., 2020c; Reis et al., 2020; Rissanen et al., 2020). Apart from CH_4_ and O_2_, it is necessary to include other physicochemical parameters that potentially influence CH_4_ oxidation. Indeed, factors such as light (Rissanen et al., 2018; Thottathil et al., 2018), phosphorus (Denfeld et al., 2018; Zhou et al., 2020), community richness (Ho et al., 2014), temperature (Yang et al., 2019), and different forms of nitrogen (Bodelier and Laanbroek, 2004) have been shown to influence CH_4_ oxidation rates. However, the impact of those environmental parameters on the methanotrophic communities is still unclear and often seems contradictory (Ho et al., 2013). Furthermore, most studies on freshwater MO neglect the potential importance of anaerobic MO and Verrucomicrobia as they are less abundant (Ho et al., 2013; Knief, 2015; Crevecoeur et al., 2017, 2019; Reis et al., 2020). As the role of rare microorganisms is still poorly understood (Galand et al., 2009), there is a pressing need to include those into the analyses for a complete understanding of the functioning and interactions in the methanotrophic community. So far, the abundance of rare MO has been associated with the expression and detection of the genes associated with the CH_4_ oxidation (Crevecoeur et al., 2017), while the diversity of the MO communities may be correlated with oxidation rates (Bodelier et al., 2013). Rare taxa can also serve as a seed for when conditions change (Graf et al., 2018; Mayr et al., 2020b) and should therefore be considered. Also, methodological issues should be taken into account as most previous studies on methanotrophs have been done using PCR-based methods looking into the diversity of 16S rRNA or *pmoA* genes (Crevecoeur et al., 2017, 2019; Rissanen et al., 2018; Mayr et al., 2020c). While being a well-established method in microbial ecology, it introduces biases to the data, especially in primer mismatches and problems related to coverage of especially new and poorly known taxa (Bourne et al., 2001; Wang et al., 2017). The shotgun metagenomic approaches are not bias free either, but they avoid the primer bias associated with amplicon sequencing. Studies looking into the ecology of methanotrophs using shotgun metagenomics are still rare (Rissanen et al., 2018; Mayr et al., 2020b). Last but not least, all known previous studies focus on one or a small number of lakes in a limited geographic area (Tsutsumi et al., 2011; Crevecoeur et al., 2017; Oswald et al., 2017; Samad and Bertilsson, 2017; Graf et al., 2018; Mayr et al., 2020c; Rissanen et al., 2020) or look only at the top layer of the studied water bodies (Crevecoeur et al., 2019), restraining the identification of factors that could be used for global estimations of the abundance and distribution of methanotrophs.

The main aims of our study were to (i) study the taxonomic patterns of MO in stratified lakes and ponds situated above 50° N of latitudes, (ii) test if environmental parameters can explain the distribution of MO groups in those water bodies, and (iii) confirm the general dominance of ƴ-MO throughout the water columns of boreal lakes and subarctic thaw ponds. To achieve these aims, we used a shotgun metagenomic dataset of 208 samples from 28 oxygen-stratified lakes and 12 permafrost thaw ponds from boreal and subarctic areas in both Europe and North America. Thus, we offer a novel insight into the diversity and distribution of methanotrophs, including rare methanotrophic taxa. While most of the studied lakes are located in Scandinavia, the addition of North American thaw ponds in the data expands our approach both geographically and functionally. Our study is based on metagenomic shotgun data and considers the importance of stratification patterns of lakes and ponds with a high concentration of dissolved organic matter (DOC).

## Materials and Methods

We obtained 208 metagenomes from four countries, covering the subarctic and boreal regions. The samples are a part of a project aiming to study microbial diversity in anoxic freshwater environments. The full details of the sample collection, sample analyses, sequencing, and data processing are provided in Buck et al. (2021). In short, for all lakes, samples were collected for both metagenome analysis and measurements of environmental parameters. For most of the lakes and ponds, the samples were taken from multiple depths, including samples from all three layers of the stratified water bodies (Figure 1). DNA was extracted from all the samples using DNeasy PowerSoil Kit (Qiagen, Hilden, Germany) following the manufacturer’s instructions. Libraries were prepared with ThruPLEX DNA-seq Prep Kit (Takara Bio Inc., Shiga, Japan) according to the manufacturer’s instructions. The protocol includes a short PCR step (seven cycles) using random primers during which sample-specific indexes are added to the samples. The shotgun sequencing of all samples was conducted at the Science for Life Laboratory (Uppsala University, Sweden) on Illumina NovaSeq6000-platform. The measured parameters varied between lakes, and for the analyses here, we selected those that were available for at least half of the samples (temperature, pH, dissolved CH_4_, O_2_, CO_2_, NH_4_, NO_3_, PO_4_, SO_4_, and Fe). For the following analyses, the samples were assigned to a layer (i.e., epi-, meta-, or hypolimnion) based on the oxygen and temperature profiles of the lakes as follows: (1) samples with O_2_ concentration above 2 mg/l were classified as epilimnion, (2) samples with temperature around 4°C and O_2_ close to 0 mg/l were classified as hypolimnion, and (3) samples from areas between the epilimnion and hypolimnion with a sharp change in oxygen and temperature were classified as metalimnion (Figure 1).

For the analyses of the methanotrophic community, we used trimmed but unassembled shotgun data, which was taxonomically classified using Kaiju with default parameters (Menzel et al., 2016) with the NCBI nr-database including eukaryotes and the fungi of JGIs 1,000 fungi project (Grigoriev et al., 2014). Kaiju is a classifier with high sensitivity and precision based on finding maximum (in-)exact matches on the protein level using the Burrows-Wheeler transform. This enabled us to detect the rare members of the community that have too low abundance to be assembled and would thus be disregarded in the analyses of assembled data (Supplementary Methods 1). The community composition was additionally analyzed using 16S rRNA reads parsed out from the shotgun data, and the community composition was compared with the Kaiju data as described in Supplementary Methods and Results.

All further analyses were done using R version 4.0.2 (R Core Team, 2020). After removal of all the reads assigned to Eukaryotes, the Kaiju data were rarefied to 90% of the number of reads in the sample with the lowest read count (1.2^∗^10^6^) in the whole dataset. We picked a value lower than the number of reads in the smallest sample to have a random subsampling for all of the samples. Rarefaction was performed using the phyloseq package in R (McMurdie and Holmes, 2013) with set.seed (1) used to initialize repeatable random subsampling. Following rarefaction, all taxa with less than 25 reads in the subsampled data were removed from the taxa table.

The abundance of methanotrophic taxa was calculated as the sum of all reads attributed to each individual taxon (e.g., MO or α-MO) divided by the sum of all reads in the sample after rarefaction. Hence, the calculated abundances are relative abundance throughout this study. The dominance of a taxon was calculated as the sum of all reads attributed to the taxon divided by the total of reads attributed to MO in the sample after rarefaction. The included taxa were the following ones: α-MO (all the bacteria in the following genera: *Methylocystis*, *Methylosinus*, *Methylocapsa*, *Methylocella*, and *Methyloferula*), ƴ-MO (all the bacteria in the order *Methylococcales*), NC10-MO [all the bacteria in genus *Ca.* Methylomirabilis)], MOA (ANME – archaea and *Ca.* Methanoperedenaceae), and V-MO (all the bacteria in the order *Methylacidiphilales*).

Non-metric multidimensional scaling (NMDS) projection and permutational multivariate analysis of variance (PERMANOVA) were performed using Bray–Curtis distance matrix and 1,000 permutations with the phyloseq package (McMurdie and Holmes, 2013). Partial least squares (PLS) regression was performed using the mixOmics package (Rohart et al., 2017) with the classic regression mode, including two components. Environmental variables were used as an observable variable (*X*) and the relative abundances of MO groups and their dominances were considered as predicted variables (*Y*). Pairwise correlations (Spearman) and Pairwise Wilcoxon rank sum tests were also performed with their *p*-values corrected using the Bonferroni method. The metagenomic dataset is available at European Nucleotide Archive (ENA) under accession number PRJEB38681.

## Results

### Gammaproteobacterial Methanotrophs Dominate the Methanotrophic Communities Throughout the Water Column

**FIGURE 2:**
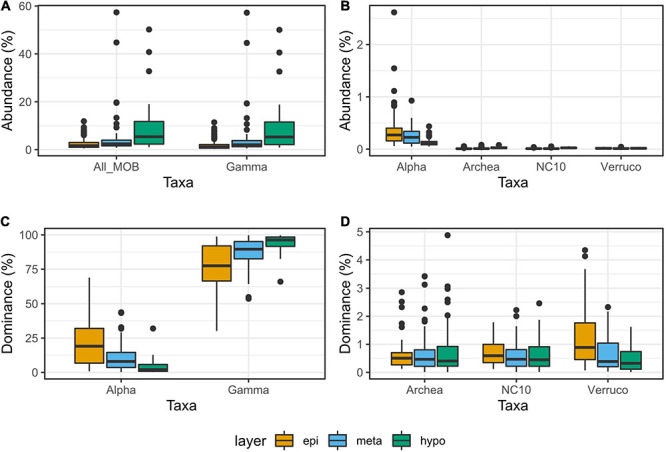
Relative abundances **(A,B)** and dominances **(C,D)** of methane oxidizers (MO) observed in the different layers of the study lakes. Abundances are the proportions of reads attributed to MO in the whole dataset. Dominances are the proportions of reads attributed to a certain MO taxon among all the reads attributed to MO. The MO taxa include: alphaproteobacterial methanotrophs (α-MO), gammaproteobacterial methanotrophs (ƴ), methanotrophic Archaea (MOA), bacterial phylum NC10 (NC10-MO), and Verrucomicrobial methanotrophs (V-MO). The lower, upper, and middle hinges correspond to the 25th, 75th, and median percentiles. The upper whisker extends up to the largest value, but not further than 1.5* the interquartile range.

The rarefied dataset was composed of 2.56^∗^10^8^ reads and included 2,822 genera. Of those reads, 1.30^∗^10^7^ (5%) were attributed to methanotrophs (MO) and classified into 26 different methanotrophic genera. The abundance of MO varied from 0.5% up to 57.4% of the reads per sample (median at 2.2%). The mean abundance of MO varied significantly between the different layers of lakes (epi-, meta-, and hypolimnion) (*p* < 0.05; Figure 2A). Highest median and mean values were found in the hypolimnion and the lowest in the epilimnion. Despite a significantly lower mean value in the metalimnion compared with the hypolimnion (*p* < 0.005), the highest abundance of MO was recorded in a sample from the metalimnion (Figure 2A).

**FIGURE 3:**
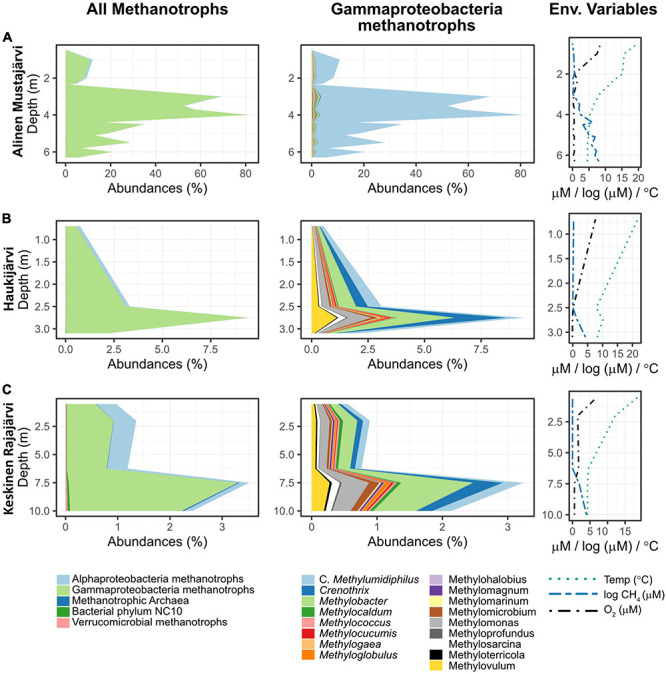
Vertical variation in the relative abundance of methane oxidizers and different genera of gammaproteobacterial methanotrophs, as well as in key environmental variables. Three lakes were selected to represent the general dominance (i.e., higher relative abundances) of gammaproteobacterial methane oxidizers **(A–C)**. Alinen Mustajärvi is an example where *Candidatus* Methylumidiphilus dominates an abundant methanotrophic community **(A)**, whereas in both Haukijärvi and Keskinen Rajajärvi, the most abundant genus in methanotrophic community is *Methylobacter* **(B,C)**. Keskinen Rajajärvi also shows a higher dominance of alphaproteobacterial methanotrophs in the epilimnion **(C)**. Please note the much higher abundance of methanotrophs in Alinen Mustajärvi **(A)**. The profiles for the rest of the lakes that had data for at least three depths are available in the Supplementary Figure 1. Note the change in scale of abundance and depth from one lake to the other.

Gammaproteobacterial methanotrophs (ƴ-MO) dominated the MO communities throughout the water columns (Figure 3 and Supplementary Figure 1) with over 50% dominance in 97% of the samples. In the rare occurrences where ƴ-MO was not the most dominant taxa, they still represented between 30 and 50% of the MO. Peaks in MO abundances were not visible in all lakes, but when existing (e.g., Alinen Mustajärvi, Figure 3A and Supplementary Figure 1), they were usually located in the metalimnion together with a fast decrease of O_2_ and increase of CH_4_ concentration. The peaks in the abundance of MO were associated with high dominance of ƴ-MO, and more specifically, of the newly discovered genus *Candidatus* Methylumidiphilus. The second most abundant group of methanotrophs, α-MO, often represented a significant proportion (i.e., dominance > 20% in 40 samples) of the methanotrophic population (e.g., Figure 3B) and in some samples could even dominate over ƴ-MO (Supplementary Figure 1). The high α-MO dominances were typically associated with the oxygenic layer.

### The Abundance of *Candidatus* Methylumidiphilus Is Correlated With the Abundance and Dominance of Gammaproteobacterial Methanotrophs

Order *Methylococcales* (i.e., ƴ-MO) was the most abundant methanotrophic taxon, representing 4.9% of all reads and 94.4% of the reads attributed to MO. This could be up to 57.2% of all reads in a single sample and up to 99.7% of reads attributed to MO (respective medians at 1.9 and 89.6%). Like the total MO community, the abundance of ƴ-MO increased toward deeper layers (*p* < 0.005) and their highest abundances were observed in metalimnion samples (Figures 2A,B). A similar pattern was observed for ƴ-MO dominance (Figures 2C,D). Furthermore, ƴ-MO dominance was strongly correlated with the total abundance of MO (ρ = 0.8). This relation between MO abundance and ƴ-MO was driven mainly by the abundance of *Candidatus* Methylumidiphilus, correlated with ƴ-MO abundance and dominance (ρ = 0.76 and 0.57). The dominance of ƴ-MO was not affected by O_2_ content or the layer of origin, and ƴ-MO dominance of over 80% could be observed in all O_2_ conditions (Figure 2C).

**FIGURE 4:**
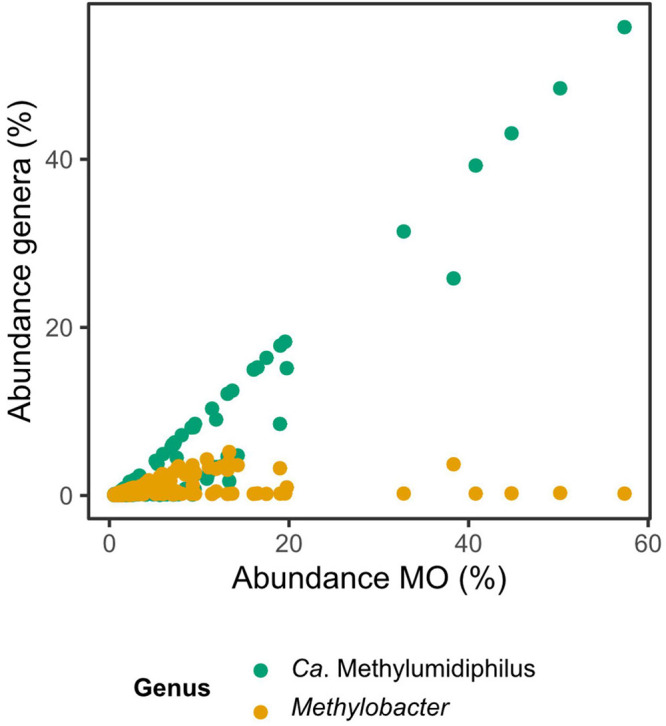
Relative abundances of the most abundant methanotrophic taxa in the function of the total relative abundance of methanotrophs (MO). The two most abundant taxa of MO are plotted against the total abundance of MO. The Spearman’s rank correlation coefficient is 0.96 for *Candidatus* Methylumidiphilus and 0.27 for *Methylobacter*. Each sample is represented by two points, one for each genus.

Among ƴ-MO, *Ca.* Methylumidiphilus accounted for 2.7% of all reads and 53.0% of reads attributed to MO. In individual samples, *Ca.* Methylumidiphilus represented up to 55.7% of all reads, corresponding to up to 97.1% of all MO reads. While *Ca.* Methylumidiphilus was the best represented MO genus in the dataset in regards to the number of samples with this taxon, *Methylobacter* was the most abundant MO in 106 samples (vs. 81 for *Ca.* Methylumidiphilus). Contrary to *Ca*. Methylumidiphilus, the relative abundance of *Methylobacter* was poorly correlated with the total abundance of MO (Figure 4). Furthermore, all samples having *Methylobacter* as the most abundant MO had a relatively low total abundance of MO (i.e., less than 20%). Other abundant ƴ-MO genera in the order of decreasing abundance were *Methylomonas*, *Crenothrix*, and *Methylovulum* (Supplementary Table 1).

**FIGURE 5:**
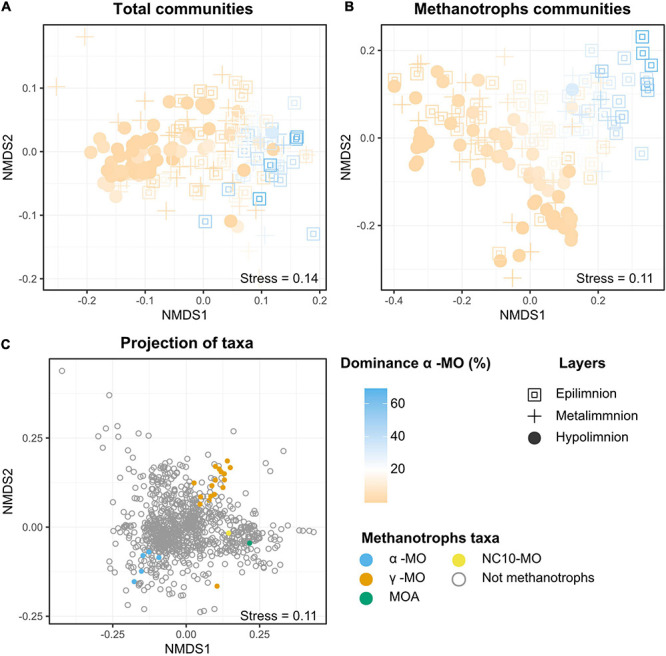
NMDS plots visualizing the composition of the **(A)** total and **(B)** methanotrophic (MO) communities in the samples as well as **(C)** distribution of different taxa. In **(A,B)**, colors represent the dominance of alphaproteobacterial methane oxidizers (i.e., the proportion of reads attributed to α-MO among all the reads attributed to MO) in each of the samples. The non-linear color scale visualizes that, except for one samples, all the samples with more than 20% of the MO communities belonging to the Alphaproteobacteria are grouped and belong to the epilimnion or the metalimnion (blue). Samples with MO communities dominated by Gammaproteobacteria (orange) are spread across the plot, including the area where Alphaproteobacteria dominated. They also include samples from all the three layers. The layers are a proxy for oxygen levels, epilimnion being fully oxic, metalimnion poor in oxygen (>0.2 mg/l), and hypolimnion anoxic. The taxa plot **(C)** shows the distance between the taxa based on their relative abundances in the samples. For readability purpose, only the 900 most abundant taxa are plotted. The MO taxa represented are alphaproteobacterial methanotrophs (α-MO), gammaproteobacterial methane oxidizers (ƴ), methanotrophic Archaea (MOA), and bacterial phylum N10 (NC10-MO).

The second most abundant class among the MO was α-MO, representing 0.2% of all reads across all the samples and 4.59% of the MO reads. The maximum abundance of α-MO was 2.6% of all reads, but α-MO could have a dominance of up to 69.0% of all the MO reads per sample. The highest abundances and dominances of α-MO were recorded in epilimnion samples and all samples but one with α-MO dominance over 20% were originating from oxic water layer (Figures 2C, 5A,B). The mean abundance of α-MO increased significantly from hypo- to metalimnion (*p* < 0.005), whereas the mean abundance between meta- and epilimnion was not significantly different (Figure 2B). Furthermore, the mean dominance of α-MO was increasing from the hypolimnion to metalimnion to epilimnion (*p* < 0.005 in all cases, Figure 2C). The most abundant α-MO genus was *Methylocystis*, followed by *Methylocapsa*, *Methylocella*, *Methylosinus*, and *Methyloferula* (Supplementary Table 1).

Anaerobic methanotrophic taxa belonging to Archaea (MOA) and bacterial phylum NC10 (NC10-MO) as well as aerobic Verrucomicrobial methanotrophs (V-MO) were each represented by a single genus: *Candidatus* Methanoperedens, *Candidatus* Methylomirabilis, and *Methylacidiphilum*, respectively. They were all detected in all samples. Overall, each of these taxa represented less than 0.02% of the total reads across all samples. Furthermore, none of these MO taxa represented more than 0.1% of all reads or 4.5% of the reads attributed to MO in one sample (Supplementary Table 1). They both had their highest mean abundances in the hypolimnion (*p* < 0.005, Figure 2B). For MOA, the difference in mean abundance between meta- and epilimnion was significant (*p* < 0.05) but not for NC10-MO. While both the maximum abundances were detected in the hypolimnion, the epi- and metalimnion abundances reached similar levels. The dominance of both MOA and NC10-MO did not vary significantly between the layers (Figure 2D). The mean normalized abundance of V-MO did not differ between layers, but its dominance was higher in the epilimnion (*p* < 0.005) than in the meta- and hypolimnion (Figures 2B,D).

### Correlation Between the Community Structure of Methanotrophs and CH_4_ Concentration Varies Between Layers

In the NMDS representation of the samples based on both the composition of the whole microbial communities and the MO communities (Figures 5A,B), samples were grouped based on their oxygen status with hypolimnion samples on one side of the plot and the epilimnion samples in the other. In contrast, the samples from metalimnion were more dispersed across the plot. However, no differences between the layers could be detected in statistical tests, likely due to the large dispersion among the oxic samples. Also, the distribution of the samples seemed to be strongly related to the dominance of α-MO. The NMDS plot based on taxa (Figure 5C) also showed that α- and ƴ-MO were grouped into two different clusters. The ƴ-MO further subdivided into three distinct clusters of genera (Supplementary Figure 2). The first cluster included genera *Methyloprofundus*, *Methylomarinum*, *Methyloglobulus*, *Methylosarcina*, *Crenothrix*, *Methylomicrobium*, *Methylomonas*, *Methylovulum*, *Methylocucumis*, and *Methylobacter*, while *Methylococcus*, *Methylogaea*, *Methylohalobius*, *Methyloterricola*, *Methylomagnum*, and *Methylocaldum*, formed the second one. The last “cluster” was composed of the genus *Ca*. Methylumidiphilus alone. The third cluster (*Ca*. *Methylumidiphilus*) was the most abundant in each layer, followed by group 1. The difference in the mean abundance between these groups was significant in each layer (*p* < 0.005).

When PLS included MO abundance (data not shown), the location of MO taxa close to the center of the PLS regression graph suggested the absence of correlation between MO abundance and the tested (i.e., environmental) variables. MO abundance was removed for further analysis because of its strong correlation with ƴ-MO. When relative abundances of the taxa were plotted, explained variances carried by the latent variables (i.e., the component axes 1 and 2) were low (<0.25). The position of the variables on the plot suggested a potential correlation between NC10-MO, MOA, CO_2_, CH_4_, Fe, PO_4_, and CO_2_, as well as between α-MO, O_2_, and temperature. ƴ-MO appeared close to the center, suggesting that none of the variables could explain its abundance (Supplementary Figure 3A). The PLS plot of MO dominances and environmental variables had latent variables explaining more of the carried variance (0.41 and 0.52 for components 1 and 2, respectively). This plot suggested a correlation between α-MO, O_2_, and temperature. Those three variables also seemed to negatively correlate with ƴ-MO dominance (Supplementary Figure 3B). The correlations between the abundances of α-MO (Supplementary Figure 4) and dominances of α-MO and ƴ-MO with O_2_ were confirmed by Spearman’s correlation (| rho| ≥ 0.5). For both MOA and NC10-MO abundances, medium or stronger correlations were confirmed with CH_4_, NH_4_, temperature, and CO_2_ but not with PO_4_ nor Fe (Supplementary Figure 5).

**FIGURE 6:**
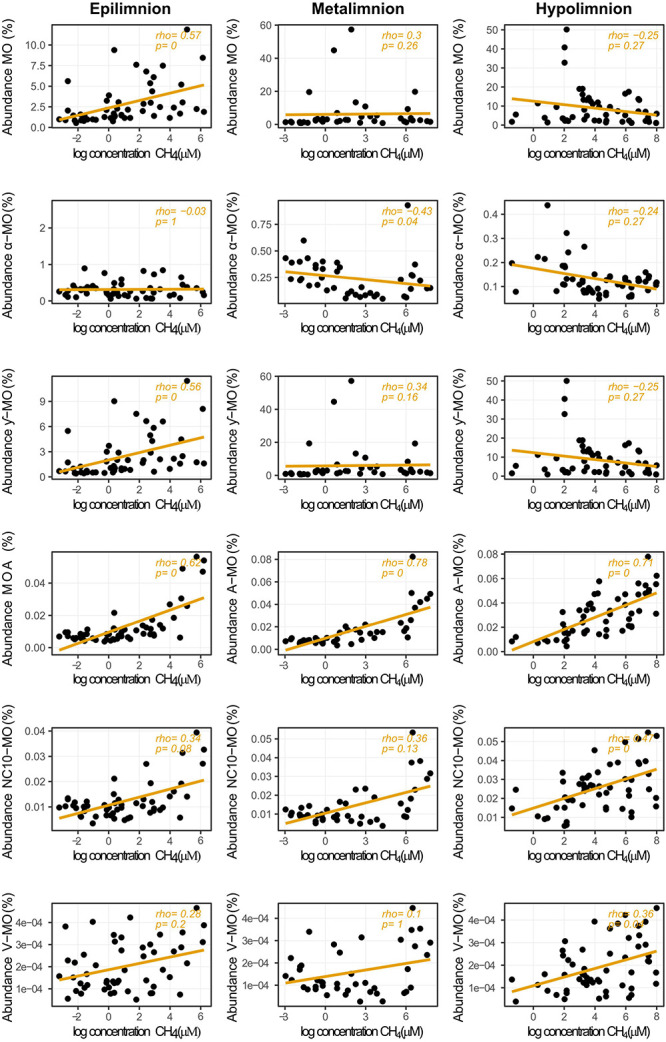
Relationship between relative abundances of methane oxidizers and CH_4_ concentrations in the different layers of stratified lakes. The taxa represented are alphaproteobacterial methanotrophs (α-MO), gammaproteobacterial methanotrophs (ƴ), Archaea (MOA), bacterial phylum NC10 (NC10-MO), Verrucomicrobial methanotrophs (V-MO), and the sum of all methane oxidizers (MO). Rho indicates Spearman’s rank correlation coefficients. *p*-values were adjusted with the Bonferroni method and rounded to two digits. Null *p*-values indicate *p* ≤ 0.005.

Whereas CH_4_ concentration seemed to have little effect on MO abundances when all samples were considered, the picture changed when we considered its effect in each layer (Figure 6). The abundance of MO in the epilimnion showed a positive medium strength correlation with CH_4_ but no clear correlation in the meta- or hypolimnion. While weak and not significant, the trend in the hypolimnion was negative. The correlation pattern observed for the ƴ-MO was the same as for MO in general. The α-MO showed a medium strength negative correlation with CH_4_ in the metalimnion. In the other layers, there were no significant correlations, but the trend was systematically negative. We did not detect any correlation between the ƴ-MO dominance and CH_4_ concentration in any of the layers.

For both MOA and NC10-MO, the correlations calculated with the entire dataset were similar to those calculated with data from each independent layer (Supplementary Tables 2–4). Relative abundances of MOA and NC10-MO were also strongly correlated (ρ = 0.78). V-MO abundance was only correlated with SO_4_ when all samples were considered together and showed medium strength correlation with O_2_ concentration in the hypolimnion (Supplementary Figure 5 and Supplementary Table 2).

## Discussion

Our multilake and multilayer approach showed that none of the measured environmental variables could predict the abundance or structure of the community, suggesting that the methanotrophs are controlled by lake-specific interactions between the methanotrophic community and environment. However, we did observe some overarching tendencies within the dataset, such as the dominance of ƴ-MO, especially the genera *Methylobacter* and *Ca*. Methylumidiphilus. In line with previous studies, our results showed a diverse methanotrophic community with variation in the abundance both across and within the different water bodies (Taipale et al., 2011; Oswald et al., 2016; Samad and Bertilsson, 2017; Crevecoeur et al., 2019). Furthermore, the pertinence of our approach based on Kaiju was confirmed by comparing our results with an alternative method based on 16S rRNA read alignment. The comparison showed high correlation between the two sets of results for the major taxa (Supplementary Methods and Results). However, the 16S-based approach was unable to detect the rare methanotrophs.

### High Relative Abundance of MO in the Meta- and Hypolimnion Has a Complex Relationship With O_2_ and CH_4_

The highest relative abundances of methanotrophs were found in the metalimnion, which is in line with previous studies that have found that MO abundance and CH_4_ oxidation are often highest in the oxygen transition zone or at the top of the hypolimnion (Kankaala et al., 2006; Tsutsumi et al., 2011; Oswald et al., 2016; Mayr et al., 2020a). However, the highest mean relative abundance of MO was found in the hypolimnion, again in line with previous studies (Oswald et al., 2015; Peura et al., 2015). The peaks in MO abundance and CH_4_ oxidation in the metalimnion have been suggested to be due to optimal O_2_ concentrations (Oswald et al., 2016; van Grinsven et al., 2020), as too high O_2_ levels may be inhibitory for MO activity (Thottathil et al., 2019). One possible explanation for the high MO abundance in the hypolimnion could be higher PO_4_ availability in anoxic conditions (Beutel et al., 2008). Indeed, several papers linked methane oxidation rates with P availability (Boiesen et al., 1993; Denfeld et al., 2016). However, our data could not confirm this possibility as no significant correlation between PO_4_ concentration and abundance was detected in any of the layers. Another reason for the high abundances of methanotrophs could be higher CH_4_ concentrations in anoxic hypolimnion, but based on our data, it does not seem that straightforward as in both meta- and hypolimnion, the highest abundances were observed in samples with lower CH_4_ concentrations (4–7 μM). Furthermore, the correlation with CH_4_ was not significant in the metalimnion and very weak and negative in the hypolimnion, suggesting that CH_4_ is not a limiting factor in those layers. The high MO abundances at low CH_4_ concentrations could reflect a rapid turnover of CH_4_ by an abundant MO community. Still, the absence of positive correlation between CH_4_ and abundance in low oxygen condition suggest that these peaks do not depend on CH_4_. In the oxic epilimnion, where CH_4_ concentrations were much lower, increases in the relative abundances of MO were observed when CH_4_ concentrations were higher. This suggests that in such conditions, CH_4_ may be a limiting factor in such conditions (Kankaala et al., 2006).

Methane being a limiting factor only when oxygen is abundant enough to be potentially inhibiting also suggests that electron acceptors could be a limiting factor for CH_4_ oxidation in the lower layers of the studied lakes. This limitation in electron acceptors in CH_4_-rich waters has been suggested by several studies where experimental addition of O_2_ or alternative electron acceptors in anoxic water as well as oxygenic photosynthesis have increased CH_4_ oxidation rates (Milucka et al., 2015; Oswald et al., 2015, 2016; van Grinsven et al., 2021). Whereas not necessarily related to abundance, these studies showed that the lack of electron acceptors could limit MO metabolism. While this would explain the lack of correlation between MO abundance and CH_4_ in low oxygen conditions and the presence of the highest recorded abundances in low oxygen samples from the metalimnion, it does not help to understand the highest mean abundance in the hypoxic layer of the lakes. These high abundances could simply be related to lower predation in anoxic waters, as zooplankton has been shown to have a strong grazing effect on MO abundance (Devlin et al., 2015). This lack of predation is also suggested by the observation of higher cell counts in the hypolimnion (Oswald et al., 2016). However, as our measurements only reflect relative abundances, augmentation in size of the whole community cannot explain the higher proportion of MO. One explanation for the higher relative abundance could be the sinking of cells following a peak in the upper layer. However, the same impact should be seen for other microbes, diluting the impact on methanotrophs. Methanotrophs could also have an advantage over other microbes as they do not need to compete for energy or carbon sources. While our data, which is based on DNA, cannot tell us if the MO in the hypolimnion are active or just the byproduct of growth in upper layers, several studies have shown that MO, both aerobic and anaerobic, can be active in the hypolimnion (Blees et al., 2014; Mayr et al., 2020b; Reis et al., 2020) and even a bloom of anaerobic MO have been observed in the hypolimnion (Graf et al., 2018). Finally, a peak of abundance in the metalimnion has been associated with stable stratification (Mayr et al., 2020a), suggesting that MO can prevail in favorable conditions. This all would suggest that the high abundance of MO in the hypolimnion is an actively growing population.

### CH_4_ Affinity Might Define the Relation Between α-Mo and ƴ-MO

The dominance of ƴ-MO has been widely reported for freshwaters (Biderre-Petit et al., 2011; Oswald et al., 2016; Rissanen et al., 2018; Chen et al., 2020; Mayr et al., 2020c), as well as higher α-MO dominance in the upper oxic layers (Biderre-Petit et al., 2011; Oswald et al., 2016; Crevecoeur et al., 2019; Mayr et al., 2020a; Reis et al., 2020). The correlation of both α-MO dominance and relative abundance with O_2_ concentration, combined with the fact that samples with high α-MO dominance all come from oxic samples, suggests that O_2_ is a crucial factor explaining the α-MO abundance and dominance. However, a closer look at our data and the literature suggests that while α-MO have higher abundance and dominance in the epilimnion, they do not appear to be responsible for the increase in MO abundance when CH_4_ concentration increases in the epilimnion. Indeed, α-MO is the only taxonomic group that does not increase in abundance with increasing CH_4_ concentration. Further, α-MO also seems to have higher abundance and dominance when CH_4_ concentrations are low in both the meta- and hypolimnion. This ability to grow in low CH_4_ concentration is in line with a well-documented high CH_4_ affinity of α-MO (Pratscher et al., 2018), particularly *Methylocystis* (Dunfield et al., 1999; Yimga et al., 2003; Knief and Dunfield, 2005; Baani and Liesack, 2008). This genus has been reported as the most abundant α-MO in an acidic boreal peat bog (Danilova et al., 2016; Esson et al., 2016) and in freshwaters (Biderre-Petit et al., 2011, 2019; Crevecoeur et al., 2019). Another hint indicating that low CH_4_ might be more critical than O_2_ in favoring α-MO is that whereas all samples with over 20% of α-MO were oxic, not all oxic samples were dominated by α-MO. Thus, several samples from the oxic environment showed the dominance of ƴ-MO, and while the inhibitory role of O_2_ on CH_4_ oxidation and its mechanisms are still unclear (Rudd et al., 1976; Thottathil et al., 2019; van Grinsven et al., 2020), it has been demonstrated that ƴ-MO can strive with high O_2_ and high CH_4_ (Hernandez et al., 2015; Oswald et al., 2015; Chu et al., 2020; Mayr et al., 2020a). Furthermore, feeding CH_4_ to an α-MO-dominated community can shift the dominance toward ƴ-MO (Knief et al., 2006; Steenbergh et al., 2009). Finally, the strong correlation between ƴ-MO abundance and dominance with the abundance of MO shows that while ƴ-MO are dominating the MO communities in most cases, this domination is getting stronger when MO abundance is high. This suggests that ƴ-MO, particularly *Candidatus* Methylumidiphilus, are fast-growing, highly competitive organisms when conditions are favorable. It, therefore, seems reasonable to see the α-MO community in boreal lakes to have a high CH_4_ affinity and slow growth rate, while the ƴ-MO has a low affinity and a fast growth rate. It could also be phrased in a more classical ecology way presenting the α-MO as stress-tolerant and ƴ-MO as competitive type, as previously suggested (Ho et al., 2013). Thus, the high α-MO dominance in aerobic samples would result from CH_4_ levels being generally lower in oxic water when the CH_4_ biofilter at the oxic-anoxic interface is particularly efficient. But the role of α-MO in limiting CH_4_ emission should not be dismissed. Due to the low affinity of ƴ>-MO for CH_4_ or methanogenesis in the epilimnion (Bogard and del Giorgio, 2016), surface water tends to be oversaturated in CH_4_ (Blees et al., 2015), leading to a release of CH_4_ to the atmosphere. In such conditions, high affinity for CH_4_ offers not only an interesting niche to exploit but is also a critical mechanism to limit CH_4_ emissions.

Both α-MO and ƴ-MO appear to have specific environmental preferences based on their lifestyle, and all genera within them seem to share similar environmental preferences. However, it is essential to notice that the separation between low CH_4_ and high O_2_—loving α-MO and high CH_4_—loving ƴ-MO is driven by a very few taxa. In addition, besides the newly discovered *Candidatus* Methylumidiphilus, which might be specific to boreal lakes (see below), in our samples, the dominating genera of both α-MO (*Methylocystis*) and ƴ-MO (*Methylobacter*, *Methylomonas*, and *Crenothrix*) are the usual suspects for freshwater CH_4_ oxidation (Biderre-Petit et al., 2011, 2019; Oswald et al., 2017; Crevecoeur et al., 2019; Mayr et al., 2020a). As all these groups of MO genera seem to share similar ecological preferences, it is tempting to assume that they also share similar preferences for CH_4_ and O_2_. Yet, it has been shown that within MO, the phylogenetic signal may be stronger for physiological traits associated with optimal growth, such as pH or temperature optimum, rather than for traits related to CH_4_ oxidation kinetics (Krause et al., 2014). This suggests that observations on preferences regarding CH_4_ concentration might only be relevant for the most abundant α- and ƴ-MO. The distribution of the other genera would then be explained by a similar preference for other variables due to phylogenetic similarity. This would be in line with our data showing the grouping of ƴ-MOB in three clusters containing closely related genera (from MO groups 1a and 1b and c, respectively) (Knief, 2015; Frindte et al., 2017; Rissanen et al., 2018).

While we argue that the affinity for CH_4_ is a key factor for explaining the niche differentiation between α- and ƴ-MO, we do not dismiss the importance of other parameters in explaining the distribution and abundance of the genera. It has been shown that at constant CH_4_ concentration, O_2_ has a selecting effect on ƴ-MO communities (Hernandez et al., 2015) and other variables like light, metals, or nitrogen compounds have had both inhibiting or enhancing effects on CH_4_ oxidation depending on the conditions (Rudd et al., 1976; Bédard and Knowles, 1989; Murase and Sugimoto, 2005; Milucka et al., 2015; Guggenheim et al., 2020). However, we could not detect any selection effect for any of the available variables. Considering that these previous studies have reported several different factors possibly regulating MO community and CH_4_ oxidation, and our lack of similar findings, it seems likely that the regulation is lake specific and depends on the specific conditions prevailing in each lake. Thus, our comparison across lakes might hide the importance of each of these parameters in individual lakes or even in lake compartments.

### Recently Described *Candidatus* Methylumidiphilus Is Globally Abundant in Boreal Lakes

Among the ƴ-MO genera, *Candidatus* Methylumidiphilus was the most abundant taxon. This abundance could be overestimated by Kaiju as the database genome of *Candidatus* Methylumidiphilus is relatively large, 6.6 Mb (*Ca*. Methylumidiphilus alinensis, GCA_003242955.1). In comparison, the average for environmental aquatic bacteria is 3.1 Mb (Rodríguez-Gijón et al., 2021). However, the observed high level of dominance seems unlikely to only be due to a methodological bias. The abundances of reads of *Ca*. Methylumidiphilus were up to two orders of magnitude higher than the abundance of the second most-abundant genus. Previously, *Ca*. Methylumidiphilus has been reported only from two boreal lakes in Southern Finland (Rissanen et al., 2018, 2020). Still, here we show that this genus is widely spread across boreal lakes and arctic thaw ponds, both in Europe and North America. Our data show that it is not only commonly found but also often represents an abundant or the most abundant member of the MO population. While *Methylobacter* was the most abundant MO in most samples, it was dominant only when the total abundance of MO was low (i.e., below 15%). This, combined with the strong correlation between *Ca.* Methylumidiphilus and MO abundance suggests that the peaks of abundances observed in certain lakes were driven by this newly described genus. *Ca.* Methylumidiphilus may therefore play an important global role in mitigating the CH_4_ emissions from the northern lakes. This could be specific to boreal and arctic lakes as other genera are known to dominate MO community in lakes sampled further south (Oswald et al., 2015, 2017; Graf et al., 2018), but the dominance of unknown OTUs (Mayr et al., 2020a) and general PCR bias makes it possible that *Candidatus* Methylumidiphilus is also present in non-boreal lakes but has escaped detection so far due to these technical problems. In fact, Rissanen et al. (2018) actually noticed that 16S rRNA gene sequences from *Ca*. M. alinensis were assigned as “unclassified Gammaproteobacteria” when using older Silva 119 (released July 24, 2014) and 123 (July 23, 2015) databases, while starting with Silva 128 database they were classified correctly as Methylococcales. This suggests that many previous 16S rRNA amplicon-based studies might have failed to correctly classify this lineage and detect it as a methanotroph. This possibility is also supported by comparing our data with the results of the 16s rRNA-based approach we used as a method validation tool. *Ca.* Methylumidiphilus was absent from the 16s rRNA reference database, whereas the abundance of unknown ƴ-MO was high (Supplementary Methods and Results).

### MOA and NC10-MO Are Potential Cooperators Throughout the Water Column

Higher MOA and NC10-MO abundances in the deeper layers, as well as their correlation with variables associated with low oxygen (CH_4_, NH_4_, PO_4_, and CO_2_), were expected as both taxa are known anaerobic CH_4_ oxidizers (Ettwig et al., 2010; Haroon et al., 2013; Vaksmaa et al., 2017). However, it might seem surprising to detect them in every sample, including those from oxic waters. Even more striking was that samples from epilimnion showed similar normalized abundances as the anoxic samples with the highest abundances of MOA and NC10-MO. Furthermore, the strong correlation observed between Archaea and NC10 abundance was consistent in every layer as well as the correlation of these two genera with CH_4_ concentration. The fact that the abundances of these two anaerobic genera increased significantly when CH_4_ concentration was high even in oxic water makes it unlikely that their presence in the epilimnion is accidental. On the contrary, it would suggest that they might be active in oxic water. While both are considered to be anaerobic organisms, they are known to be the least O_2_ tolerant (Guerrero-Cruz et al., 2018) and potentially get more active when O_2_ is added to anoxic media (Kampman et al., 2018). They might also benefit from the higher concentration of NO_3_ in aerobic conditions. A similar kind of activity of an anaerobic organism in oxic environment has been suggested for methanogenic Archaea (Bogard and del Giorgio, 2016). This may be facilitated by anoxic microniches inside particles (Schramm et al., 1999; Lehto et al., 2014). The sharp increase in the abundance of MOA and NC10-MO in samples with CH_4_ concentration over 5 μM in the epi- and metalimnion suggest that they need high CH_4_ to strive in oxic conditions. However, as abundances of anaerobic methanotrophs at low CH_4_ concentrations are higher in anoxic conditions, it seems more likely that the sharp rise observed in the upper layer is related to the inhibitory effect of O_2_. Higher CH_4_ concentrations could compensate O_2_ limited affinity for CH_4_ or reflect the presence of more favorable conditions for anoxic organisms (e.g., anoxic microniches). The strong correlation between the abundance of the two taxa suggests a cooperative interaction between them. Indeed, the most abundant of them, *Ca.* Methanoperedens uses NO_3_ as an electron acceptor and releases NO_2_ that can be used by *Ca*. Methylomirabilis. This possibility is supported by CH_4_-fed enrichment that coselected both *Ca.* Methanoperedens and *Ca*. Methylomirabilis when NO_3_ was the only electron acceptor provided (Vaksmaa et al., 2017; Gambelli et al., 2018).

## Conclusion

Our study represents the first large-scale analysis of methanotrophic communities from oxygen-stratified lakes spanning from Europe to North America. While most of our data come from Scandinavian lakes, the presence of North American ponds suggests a similar pattern for this region. With these data, we confirmed that many of the results gained from analyzing a limited number of lakes are relevant for freshwater bodies above 50°N. Furthermore, we suggest that the ability to consume CH_4_ at a low concentration is probably a key element in discriminating between the dominance of α-MO and ƴ-MO. The first appears to be more stress tolerant with a high affinity for CH_4_ and low growth speed, while the latter are strong competitors with low affinity and high growth rate.

Consequently, α-MO dominate the communities when MO represent only a small fraction of the microbiome in the surface layer characterized by low CH_4_ concentration. When CH_4_ is not a limiting resource, ƴ-MO not only dominate the MO communities but potentially the whole microbiome. The high affinity of α-MO suggests that despite having lower abundances than ƴ-MO, they could play an important role in consuming CH_4_ when concentration are not suitable for fast-growing ƴ-MO, and α-MO could have a significant role in diminishing the emissions from the recently suggested CH_4_ production in the oxic water column (Bogard and del Giorgio, 2016; Günthel et al., 2020; Li et al., 2020). Thus, while representing low abundance organisms, the α-MO could be critical for limiting CH_4_ emissions from lakes as CH_4_ oversaturation of oxic layer is a widespread phenomenon (Blees et al., 2015; León-Palmero et al., 2020). Among ƴ, *Candidatus* Methylumidiphilus was found in all lakes and appeared to be the genus responsible for peaks of the relative abundance of MO. Therefore, it is suggested to play an important role in diminishing the CH_4_ emissions from the boreal lakes and arctic thaw ponds. Overall, our results significantly improve our knowledge on the diversity and abundance of methanotrophs and strongly suggest that the abundance and diversity of the methanotrophs in any single lake are strongly dependent on specific conditions of that particulate lake. Thus, these communities are controlled by local rather than global factors.

## Data Availability Statement

Publicly available datasets were generated for this study. This data can be found here: https://www.ncbi.nlm.nih.gov/bioproject/PRJEB38681.

## Author Contributions

GM, AR, MB, SG, and SP designed the study. GM carried out data analyses with help from MM. GM was responsible for data interpretation with regular input of AR, SG, and SP. All authors discussed the results and conclusion. GM led manuscript writing. All authors participated with substantial comments and edits of the manuscript.

## Conflict of Interest

The authors declare that the research was conducted in the absence of any commercial or financial relationships that could be construed as a potential conflict of interest.

## Publisher’s Note

All claims expressed in this article are solely those of the authors and do not necessarily represent those of their affiliated organizations, or those of the publisher, the editors and the reviewers. Any product that may be evaluated in this article, or claim that may be made by its manufacturer, is not guaranteed or endorsed by the publisher.
