# Supplementary material for: Candidatus Methylumidiphilus Drives Peaks in Methanotrophic Relative Abundance in Stratified Lakes and Ponds Across Northern Landscapes
Source: Front Microbiol. 2021 Aug 12;12:669937. doi: 10.3389/fmicb.2021.669937 (PMC8397446; doi:10.3389/fmicb.2021.669937)
Supplement: Supplementary file 1 [file Data_Sheet_1.docx]

***Supplementary material***

**Supplementary Table S1**: **Summary of relative abundances and dominances of all methane oxidizer (MO) genera present in our data set**. Abundances are the proportions of reads attributed to MO in the whole data set. Dominances are the proportions of reads attributed to a certain MO taxon among all the reads attributed to MO.

| **Taxa** | **Mean abundance** | **Median abundance** | **Max abundance** | **Mean dominance** | **Median dominance** | **Max dominance** |
| --- | --- | --- | --- | --- | --- | --- |
| *Methylumidiphilus* | 2.678 | 0.281 | 55.724 | 25.583 | 12.911 | 97.140 |
| *Methylobacter* | 0.691 | 0.344 | 5.177 | 19.147 | 20.523 | 45.208 |
| *Methylomonas* | 0.369 | 0.224 | 3.222 | 11.582 | 11.394 | 51.166 |
| *Crenothrix* | 0.265 | 0.120 | 2.332 | 7.156 | 7.079 | 33.503 |
| *Methylovulum* | 0.221 | 0.104 | 1.441 | 5.756 | 5.772 | 20.021 |
| *Methylomicrobium* | 0.089 | 0.054 | 0.607 | 2.741 | 2.898 | 7.178 |
| *Methylosarcina* | 0.088 | 0.057 | 0.469 | 2.838 | 2.776 | 7.136 |
| *Methylocystis* | 0.085 | 0.055 | 0.618 | 4.507 | 2.905 | 25.940 |
| *Methylocaldum* | 0.074 | 0.045 | 0.948 | 2.164 | 2.084 | 8.347 |
| *Methyloglobulus* | 0.053 | 0.030 | 0.330 | 1.605 | 1.709 | 3.711 |
| *Methylocapsa* | 0.051 | 0.029 | 0.729 | 2.637 | 1.478 | 19.249 |
| *Methyloterricola* | 0.050 | 0.024 | 1.047 | 1.200 | 1.124 | 3.759 |
| *Methylomagnum* | 0.047 | 0.028 | 0.565 | 1.412 | 1.259 | 8.119 |
| *Methylocella* | 0.044 | 0.021 | 1.393 | 2.164 | 1.066 | 36.773 |
| *Methylococcus* | 0.042 | 0.027 | 0.376 | 1.479 | 1.114 | 14.908 |
| *Methylocucumis* | 0.037 | 0.017 | 0.288 | 1.000 | 0.992 | 4.722 |
| *Methylosinus* | 0.034 | 0.024 | 0.178 | 1.694 | 1.205 | 9.582 |
| *Methylomarinum* | 0.024 | 0.015 | 0.137 | 0.797 | 0.809 | 1.695 |
| *Methanoperedens* | 0.020 | 0.015 | 0.082 | 0.881 | 0.589 | 4.880 |
| *Methyloferula* | 0.018 | 0.012 | 0.101 | 0.971 | 0.565 | 7.276 |
| *Methyloprofundus* | 0.018 | 0.010 | 0.105 | 0.550 | 0.556 | 1.504 |
| *Methylomirabilis* | 0.016 | 0.013 | 0.055 | 0.685 | 0.610 | 2.460 |
| *Methylacidiphilum* | 0.016 | 0.012 | 0.047 | 0.773 | 0.605 | 4.348 |
| *Methylogaea* | 0.011 | 0.008 | 0.115 | 0.362 | 0.353 | 0.982 |
| *Methylohalobius* | 0.008 | 0.007 | 0.170 | 0.316 | 0.280 | 1.420 |

**Supplementary Table S2:** Spearman’s rank correlation coefficients (rho) between the abundances and environmental variables in the epilimnion. All p-values (p) were corrected using the Bonferroni method.

|  | | | | | | | | | | | | |
| --- | --- | --- | --- | --- | --- | --- | --- | --- | --- | --- | --- | --- |
|  | | | | | | | | | | | | |
|  | **MO** | | **α-MO** | | **ƴ-MO** | | **MOA** | | **NC10-MO** | | **V-MO** | |
|  | **rho** | **p** | **rho** | **p** | **rho** | **p** | **rho** | **p** | **rho** | **p** | **rho** | **p** |
| *O_2_* | -0.37 | 0.19 | 0.13 | 1.00 | -0.47 | 0.01 | -0.36 | 0.28 | -0.39 | 0.11 | -0.16 | 1.00 |
| *Temperature* | -0.34 | 0.35 | 0.29 | 1.00 | -0.42 | 0.03 | -0.29 | 1.00 | -0.45 | 0.01 | -0.06 | 1.00 |
| *CH_4_* | 0.57 | 0.00 | -0.03 | 1.00 | 0.56 | 0.00 | 0.62 | 0.00 | 0.34 | 0.77 | 0.28 | 1.00 |
| *CO_2_* | 0.32 | 1.00 | -0.40 | 0.20 | 0.36 | 0.49 | 0.28 | 1.00 | 0.34 | 0.85 | -0.04 | 1.00 |
| *pH* | -0.08 | 1.00 | -0.70 | 0.00 | 0.09 | 1.00 | -0.21 | 1.00 | 0.02 | 1.00 | -0.20 | 1.00 |
| *NH_4_* | 0.25 | 1.00 | -0.34 | 1.00 | 0.25 | 1.00 | 0.23 | 1.00 | 0.28 | 1.00 | -0.09 | 1.00 |
| *NO_3_* | -0.28 | 1.00 | -0.06 | 1.00 | -0.41 | 0.68 | -0.56 | 0.02 | -0.47 | 0.21 | -0.62 | 0.00 |
| *PO_4_* | 0.18 | 1.00 | 0.37 | 1.00 | 0.08 | 1.00 | -0.07 | 1.00 | 0.10 | 1.00 | 0.15 | 1.00 |
| *SO_4_* | -0.02 | 1.00 | -0.47 | 0.19 | 0.03 | 1.00 | -0.22 | 1.00 | -0.07 | 1.00 | -0.58 | 0.01 |
| *Fe* | -0.10 | 1.00 | 0.15 | 1.00 | -0.15 | 1.00 | 0.17 | 1.00 | -0.03 | 1.00 | 0.02 | 1.00 |

**Supplementary Table S3:**  Spearman’s rank correlation coefficients (rho) between the abundances and environmental variables in the metalimnion. All p-values (p) were corrected using the Bonferroni method.

|  | | | | | | | | | | | | |
| --- | --- | --- | --- | --- | --- | --- | --- | --- | --- | --- | --- | --- |
|  | | | | | | | | | | | | |
|  | **MO** | | **α-MO** | | **ƴ-MO** | | **MOA** | | **NC10-MO** | | **V-MO** | |
|  | **rho** | **p** | **rho** | **p** | **rho** | **p** | **rho** | **p** | **rho** | **p** | **rho** | **p** |
| *O2* | -0.12 | 1 | 0.32 | 1.00 | -0.16 | 1 | -0.24 | 1.00 | -0.16 | 1.00 | -0.15 | 1.00 |
| *Temperature* | -0.02 | 1 | 0.26 | 1.00 | -0.04 | 1 | -0.36 | 0.98 | -0.25 | 1.00 | -0.15 | 1.00 |
| *CH4* | 0.30 | 1 | -0.43 | 0.25 | 0.34 | 1 | 0.78 | 0.00 | 0.36 | 1.00 | 0.10 | 1.00 |
| *CO2* | 0.31 | 1 | -0.26 | 1.00 | 0.33 | 1 | 0.75 | 0.00 | 0.55 | 0.01 | 0.19 | 1.00 |
| *pH* | -0.27 | 1 | -0.37 | 1.00 | -0.27 | 1 | 0.50 | 0.74 | 0.54 | 0.37 | -0.13 | 1.00 |
| *NH4* | 0.15 | 1 | -0.36 | 1.00 | 0.16 | 1 | 0.58 | 0.02 | 0.42 | 0.75 | 0.06 | 1.00 |
| *NO3* | -0.17 | 1 | -0.29 | 1.00 | -0.14 | 1 | -0.44 | 0.60 | -0.55 | 0.06 | -0.58 | 0.02 |
| *PO4* | -0.21 | 1 | 0.02 | 1.00 | -0.21 | 1 | -0.01 | 1.00 | -0.29 | 1.00 | -0.22 | 1.00 |
| *SO4* | -0.06 | 1 | -0.44 | 0.27 | -0.01 | 1 | 0.12 | 1.00 | -0.15 | 1.00 | -0.60 | 0.00 |
| *Fe* | -0.40 | 1 | -0.09 | 1.00 | -0.38 | 1 | 0.36 | 1.00 | -0.06 | 1.00 | -0.07 | 1.00 |

**Supplementary Table S4:** Spearman’s rank correlation coefficients (rho) between the abundances and environmental variables in the hypolimnion. All p-values (p) were corrected using the Bonferroni method.

|  | | | | | | | | | | | | |  |
| --- | --- | --- | --- | --- | --- | --- | --- | --- | --- | --- | --- | --- | --- |
|  | | | | | | | | | | | | |  |
|  |  |  |  |  |  |  |  |  |  |  |  |  | |
|  | **MO** | | **α-MO** | | **ƴ-MO** | | **MOA** | | **NC10-MO** | | **V-MO** | |  |
|  | **rho** | **p** | **rho** | **p** | **rho** | **p** | **rho** | **p** | **rho** | **p** | **rho** | **p** | |
| *O2* | 0.14 | 1.00 | 0.03 | 1.00 | 0.12 | 1.00 | 0.40 | 0.15 | 0.32 | 0.91 | 0.52 | 0.00 | |
| *Temperature* | -0.37 | 0.35 | 0.28 | 1.00 | -0.35 | 0.51 | -0.58 | 0.00 | -0.57 | 0.00 | -0.32 | 1.00 | |
| *CH4* | -0.25 | 1.00 | -0.24 | 1.00 | -0.25 | 1.00 | 0.71 | 0.00 | 0.47 | 0.02 | 0.36 | 0.42 | |
| *CO2* | -0.39 | 0.20 | -0.34 | 0.64 | -0.37 | 0.29 | 0.42 | 0.08 | 0.15 | 1.00 | -0.08 | 1.00 | |
| *pH* | 0.26 | 1.00 | -0.64 | 0.02 | 0.30 | 1.00 | 0.07 | 1.00 | 0.11 | 1.00 | -0.28 | 1.00 | |
| *NH4* | 0.02 | 1.00 | -0.32 | 1.00 | 0.04 | 1.00 | 0.58 | 0.00 | 0.51 | 0.02 | 0.29 | 1.00 | |
| *NO3* | 0.29 | 1.00 | 0.16 | 1.00 | 0.29 | 1.00 | -0.40 | 0.41 | -0.18 | 1.00 | 0.02 | 1.00 | |
| *PO4* | -0.13 | 1.00 | 0.30 | 1.00 | -0.15 | 1.00 | -0.10 | 1.00 | 0.08 | 1.00 | 0.32 | 1.00 | |
| *SO4* | 0.07 | 1.00 | -0.36 | 0.46 | 0.09 | 1.00 | -0.25 | 1.00 | -0.15 | 1.00 | -0.46 | 0.03 | |
| *Fe* | -0.39 | 0.64 | -0.15 | 1.00 | -0.40 | 0.57 | 0.17 | 1.00 | 0.02 | 1.00 | -0.04 | 1.00 | |
|  |  |  |  |  |  |  |  |  |  |  |  |  | |


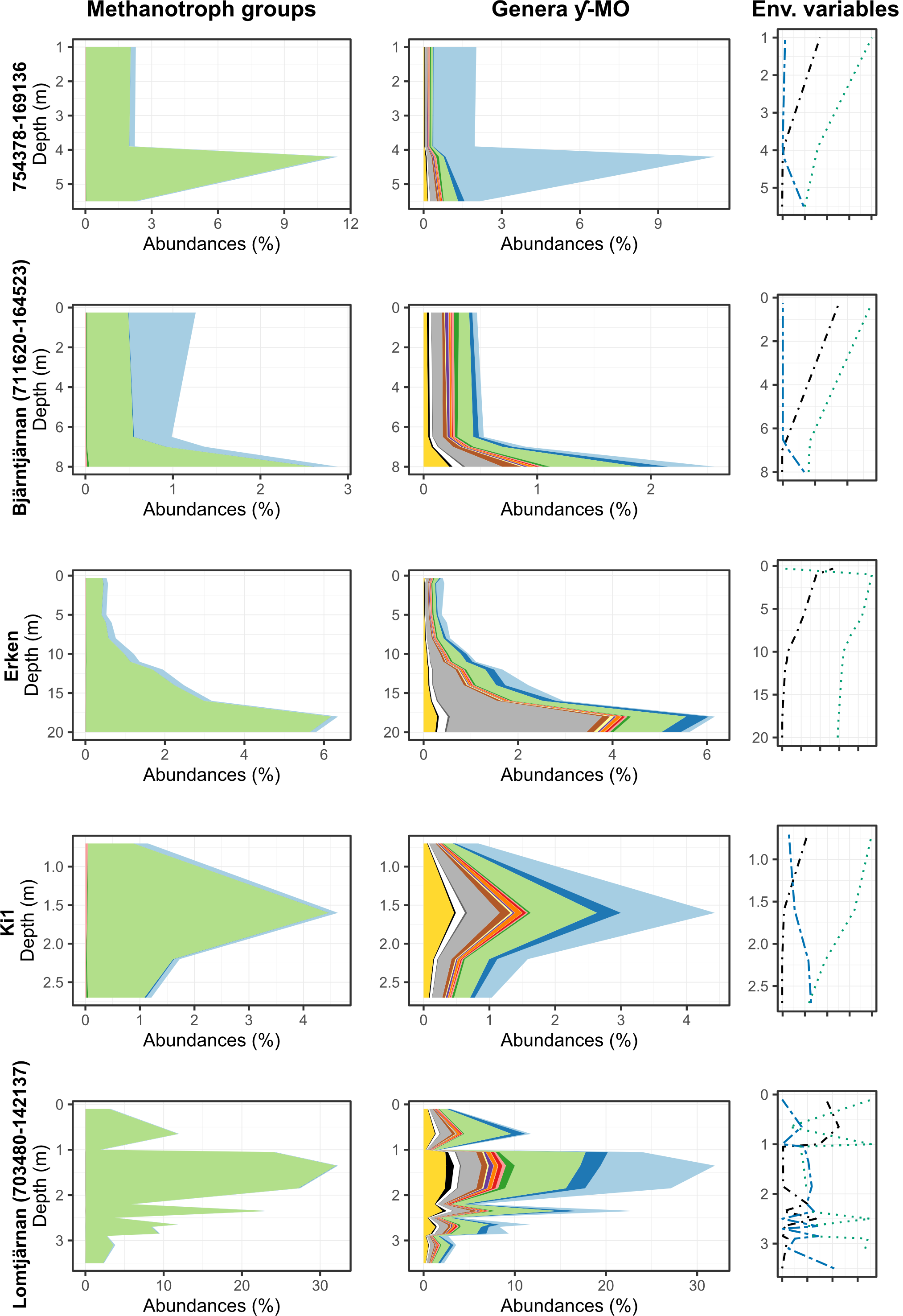


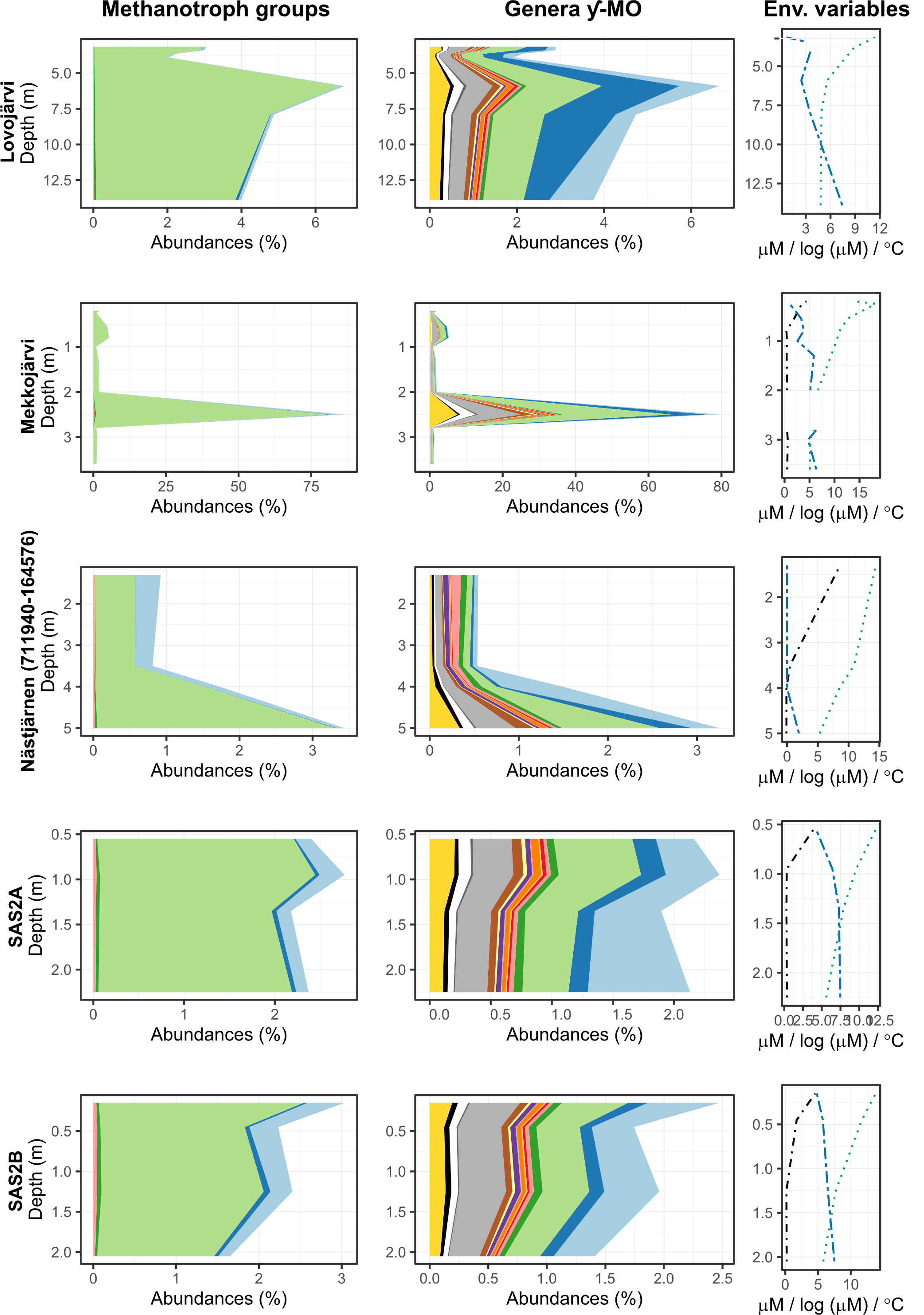

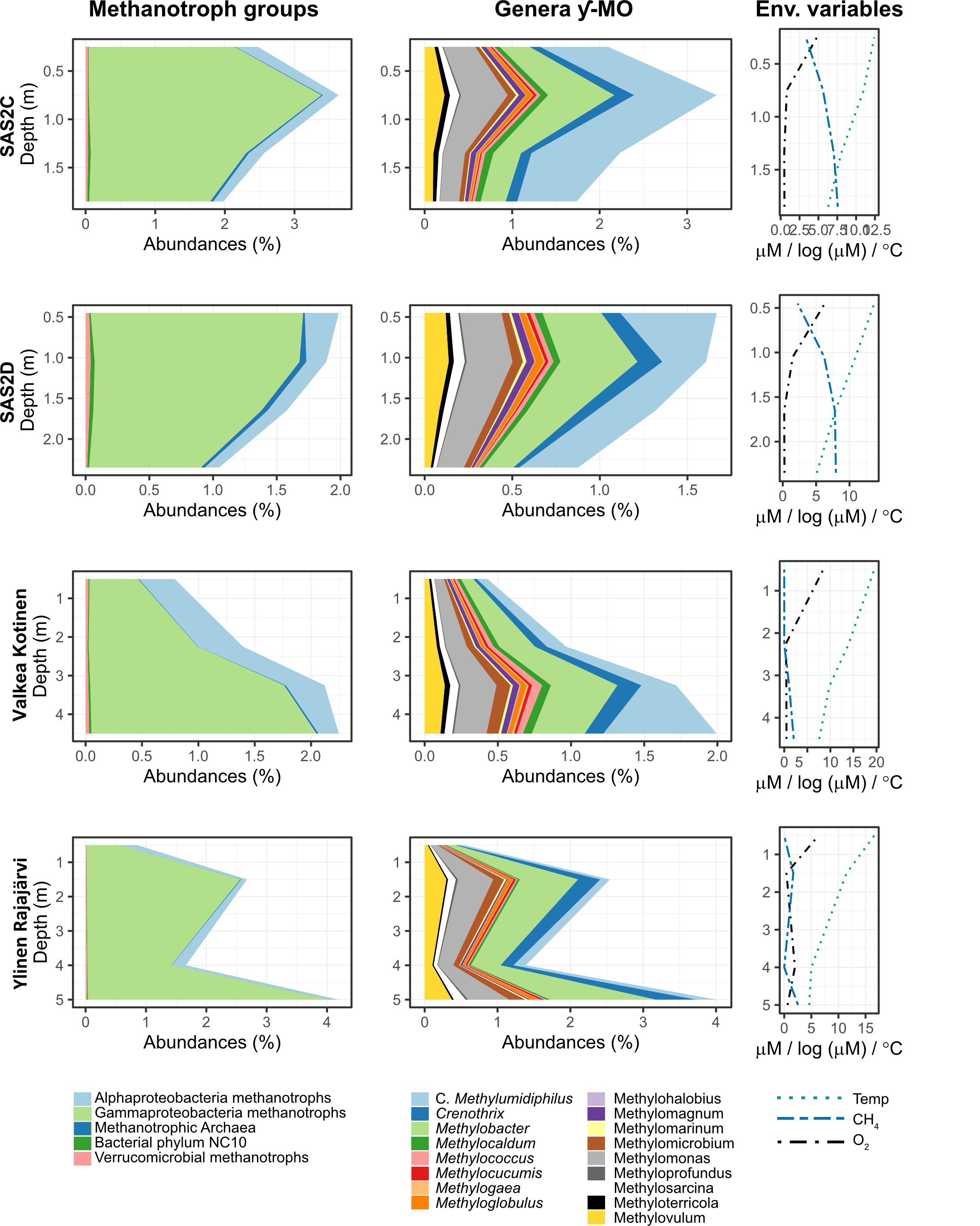


**Supplementary Figure S1. Vertical variation in the relative abundances of methane oxidizers (MO) (left column) and different genera of gammaproteobacterial methanotrophs (middle column), as well as in key environmental (Env.) variables (CH_4_ and O_2_ concentration and temperature; right column).** Profiles are presented for all the lakes that have data collected from at least three depths. Note the change in the scale of abundance and depth from one lake to another.

**
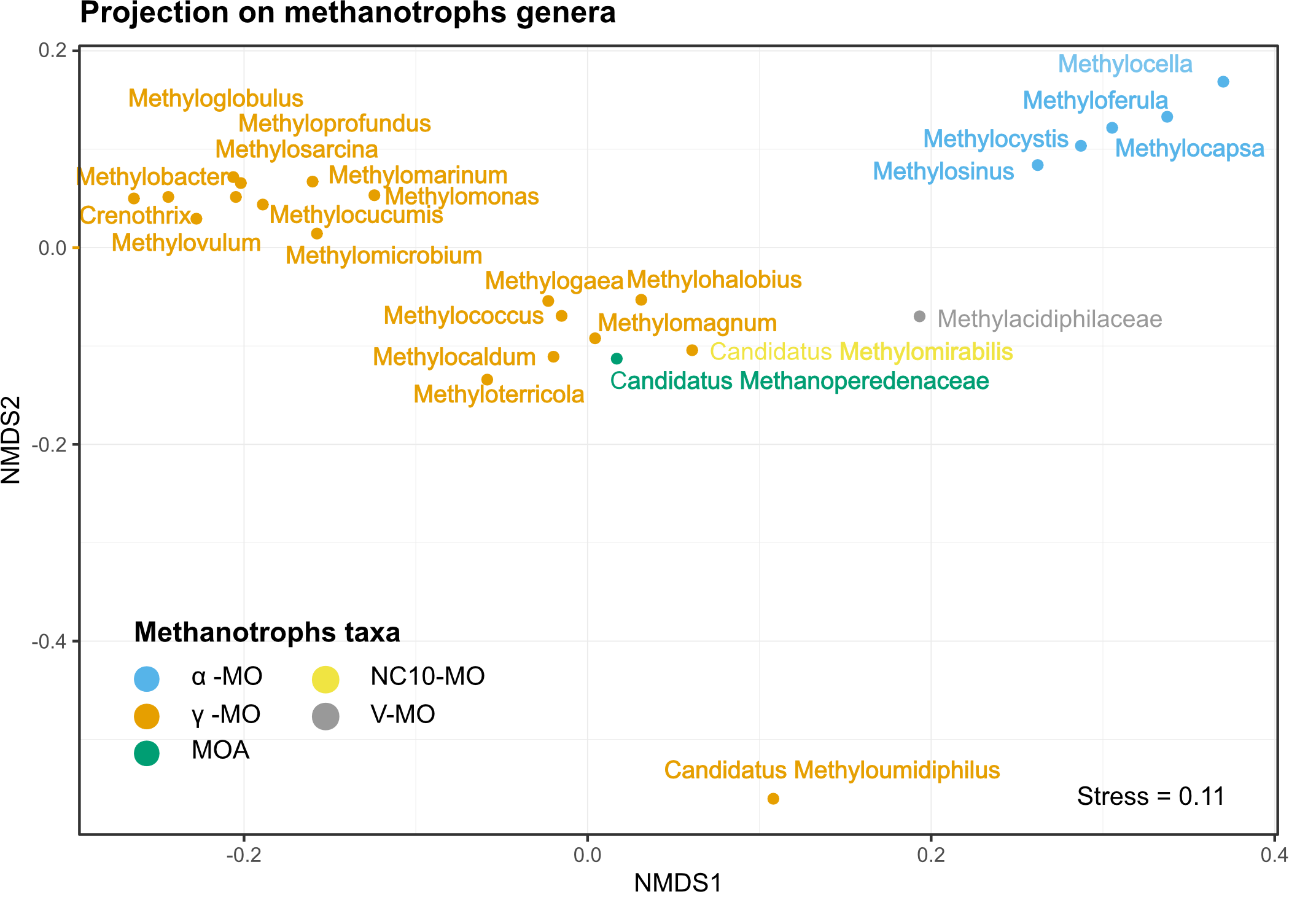
**

**Supplementary Figure S2**. **Non-metric multidimensional scaling** **plot (NMDS) of the methanotrophic (MO) genera.** NMDS representing Bray-Curtis dissimilarities between MO taxa based on their relative abundances in each of the samples. The Bray-Curtis dissimilarities matrix was calculated for the abundances of MO genus only. Abbreviations for the taxonomic groups are: alphaproteobacterial methanotrophs (α-MO), gammaproteobacterial methanotrophs (ƴ-MO), archaeal methanotrophs (MOA), bacterial phylum NC10 (NC10-MO), and Verrucomicrobial methanotrophs (V-MO).

**
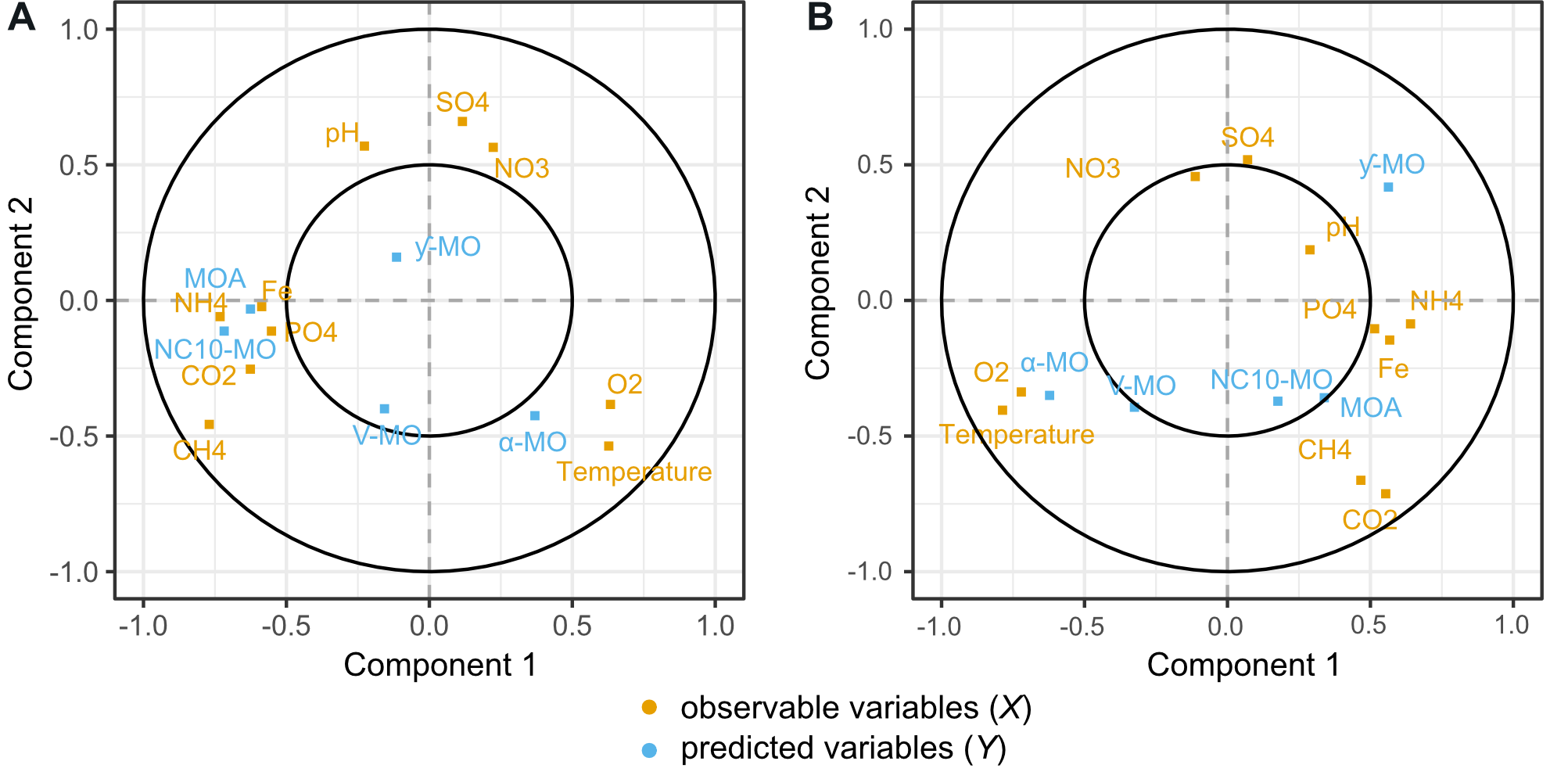
**

**Supplementary Figure S3. Correlation plot of the Partial Least Squares (PLS) regression analysis to evaluate the potential influence of environmental variables on (A) the relative abundance and (B) the dominance of methane oxidizers (MO).** Components axis represent latent variables (i.e. a linear combinations of the original observed variables). Position of the observed variables (*X*) indicates how much each *X* variables explains the component axis. Positions of the predicted variables (*Y)* indicate how much of each *Y* variables variance is carried by the latent variable. A variable (*X* or *Y*) that stands right on an axis is only explains (*X)*/ is explained (*Y*) by that latent variable. The distance to the axis indicates the strength of the correlation between the variables (*X or Y*) and the latent variables (component 1 or 2). Consequently, the angles between variables indicate if they are correlated to each other. A null or 180° angle suggest a strong correlation whereas a 90° angle excludes the possibility of a correlation. The strength of the actual correlation can be attenuated by the distance to the center and by the explained variances carried by each component axis. Explained variances carried by the latent variables were 0.25 (comp1) and 0.16 (comp 2) for *X* and 0.41 (comp1) and 0.52 (comp 2) for *Y*.

Abundances are the proportions of reads attributed to MO in the whole set of reads. Dominances are the proportion of reads attributed to a certain MO taxon among all the reads attributed to MO. The MO taxa included to the analysis are gammaproteobacterial methane oxidizers (ƴ-MO), alphaproteobacterial methanotrophs (α-MO), methanotrophic Archaea (MOA), bacterial phylum NC10 (NC10-MO), and Verrucomicrobial methanotrophs (V-MO).


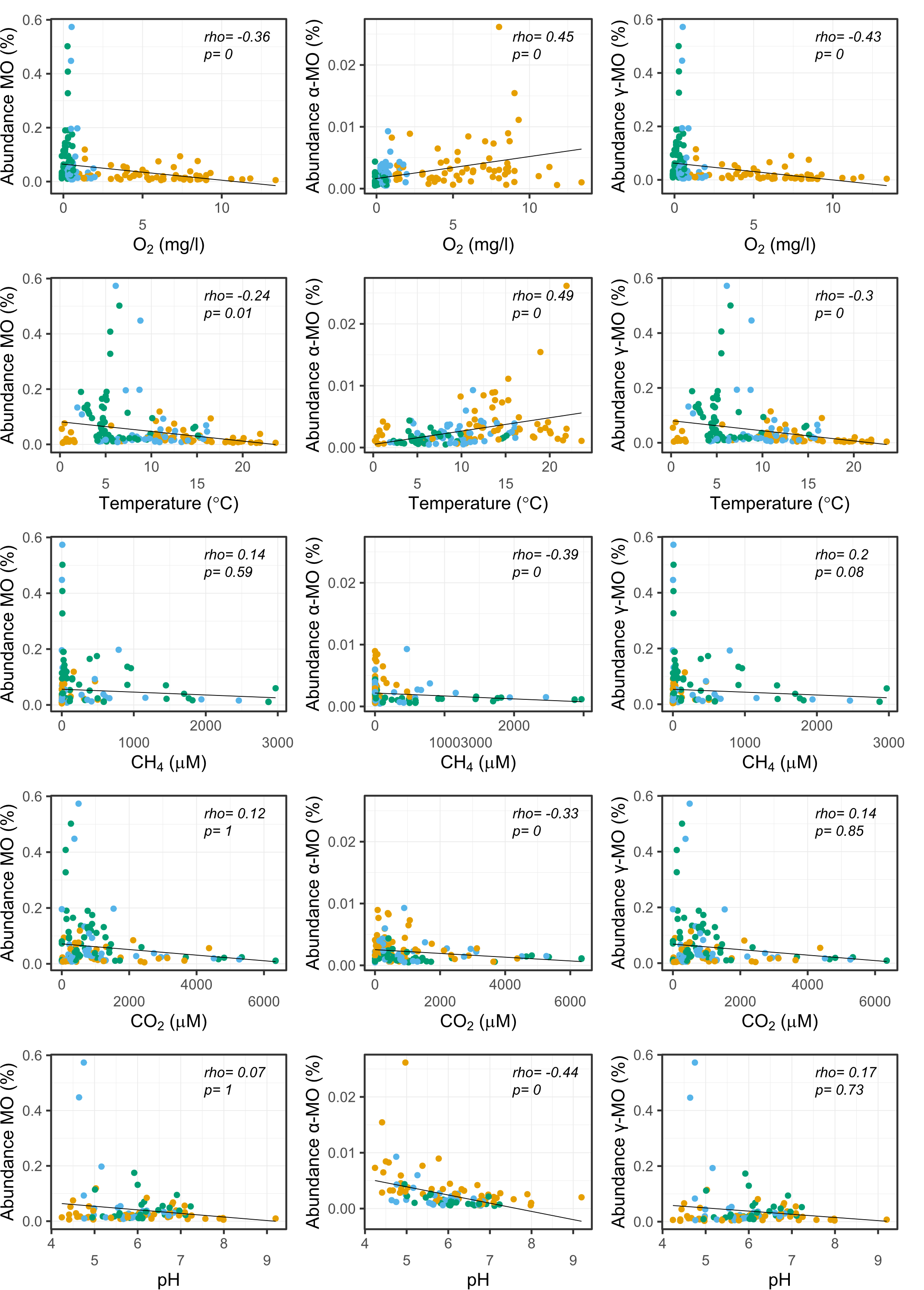


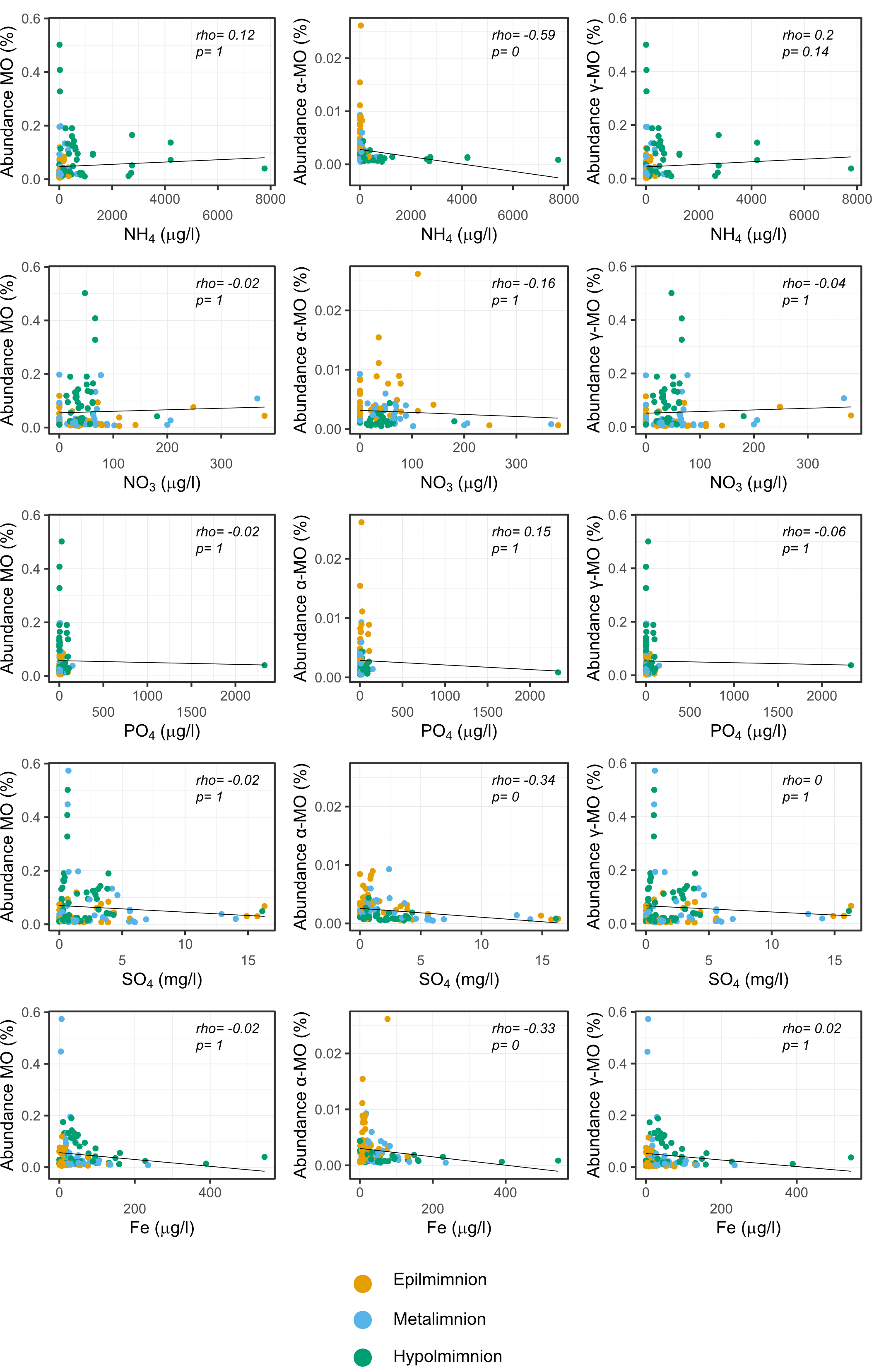


**Supplementary Figure S4. Relationships between the environmental variables and the relative abundances of the most abundant methane oxidizers (MO).** The plots include the alphaproteobacterial methanotrophs (α-MO), gammaproteobacterial methane oxidizers (ƴ-MO) and the total methanotrophic community (MO). Rho indicates Spearman's rank correlation coefficients. P-values were adjusted for multiple comparisons using the Bonferroni method. Null p-values indicate p≤0.005.


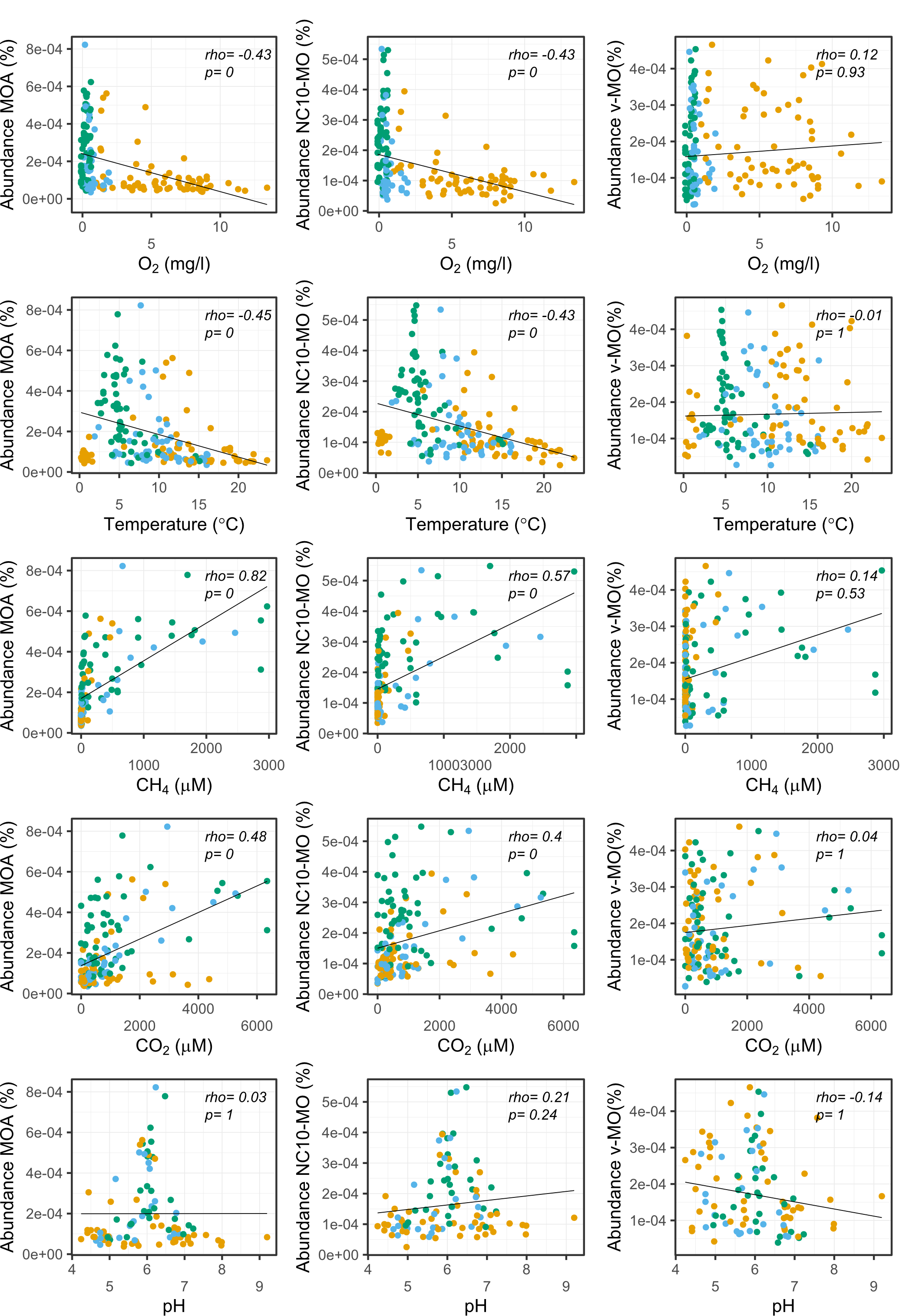


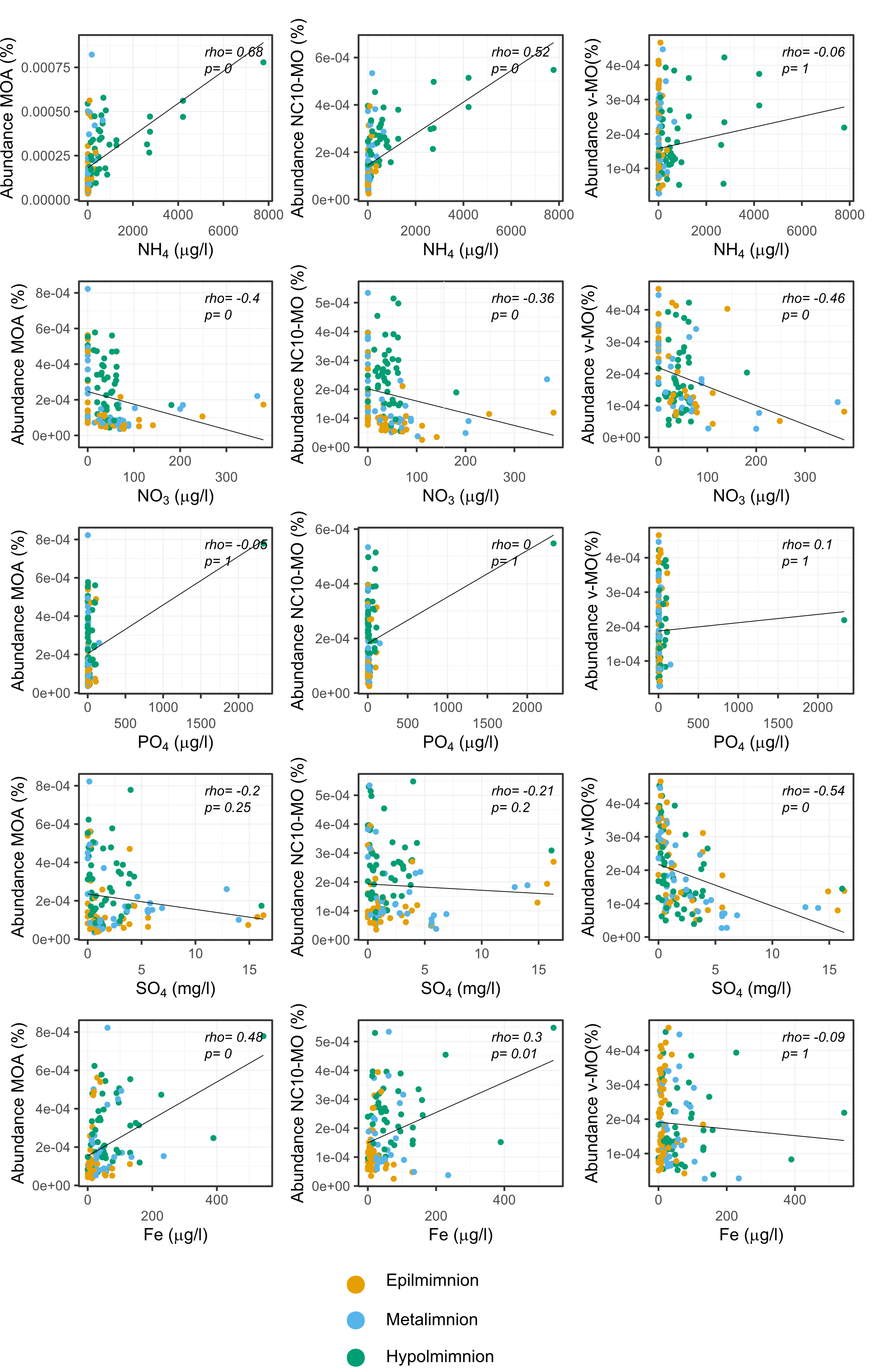


**Supplementary Figure S5. The relationships between the environmental variables and the relative abundances of the rare methane oxidizers.** The plots include archaeal methanotrophs (MOA), bacterial phylum NC10 (NC10-MO), and verrucomicrobial methanotrophs (V-MO). Rho indicates Spearman's rank correlation coefficients. P-values were adjusted for multiple comparisons using the Bonferroni method and rounded to two digits. Null p-values indicate p≤0.005.

**Supplementary Methods and Results: Comparison of relative abundances obtained with alternative method**

*Methods*

To confirm the results obtained with Kaiju, we also extracted 16S rRNA genes from the metagenomes. For this purpose, a subset of 10 million read was separated for each of metagenomic samples. Reads affiliated to ribosomal RNA genes (16S/18S) were detected in these subsets using SSU-ALIGN software (Nawrocki, 2009). Putative prokaryotic 16S rRNA sequences were compared against the SILVA reference database (release 132SSUParc) using BLAST. Taxonomic affiliations of the reads were assigned based on their closest hit if the read was ≥ 90bp, at the similarity threshold of ≥ 90. Relative abundances calculated with the Kaiju data were then compared with the relative abundances of putative prokaryotic 16S rRNA sequences assigned for the same taxa. The SILVA database does not have a 16S rRNA sequence of *Ca.* Methylumidiphilus, thus it is frequently assigned as unclassified ƴ-MO (Rissanen et al., 2018). For the analyses here, we used unclassified ƴ-MO as an indicator for *Ca.* Methylumidiphilus.

*Results*

A total of 1166193 reads were assigned to putative prokaryotic 16S rRNA sequences. Number of assigned reads for the samples ranged from 246 to 11637, with mean and median values of 5399 and 5416 respectively. Among these reads, 63597 (5.5 %) were attributed to MO. The relative abundances of MOs in the samples varied from 0.1 % to 46 %.

Relative abundances of total MO and ƴ-MO were strongly correlated between the Kaiju and 16S rRNA gene read data sets (rho >0.8) and values were in the same order of magnitude. It also appeared that the relation between the two data sets changed depending on what taxa dominated the MO community (Supplementary Figure SM1A and SM1B). This indicates that while very different in their approach (Kaiju is protein-level classifier, whereas the validation methods is based on ribosomal RNA genes) the two methods give similar results. The difference in slope for samples depending on their most dominant ƴ-MO suggests, that Kaiju could overestimate MO in samples dominated by *Ca.* Methylumidiphilus or underestimate them in samples dominated by Methylobacter. Or alternatively, the 16S rRNA method was under- and overestimating these, respectively. For Kaiju the estimates could be affected by the relatively large size of *Ca.* Methylumidiphilus genome (6.6 Mb). However, since there is no reference for *Ca.* Methylumidiphilus in the Silva database, this is the natural cause of lack of observations and the algorithm is likely to dismiss some reads that otherwise would have been counted as ƴ-MO. However, interestingly not all samples dominated by *Ca.* Methylumidiphilus showed signs of a potential overestimation by Kaiju. This suggests that the over/under estimation observed could be the result a combination of the two explanations or that other variables could be at play. Despite this difference, the two methods clearly yield comparable results when it comes to the total abundances of MO and ƴ-MO. Importantly, despite the lack of *Ca.* Methylumidiphilus 16S rRNA sequence in the database, the 16S rRNA gene approach confirmed that the highest relative abundances of MO are recorded in samples dominated by *Ca.* Methylumidiphilus.

As stated, 16S rRNA gene sequences of *Ca.* Methylumidiphilus were absent in the Silva database at the time of this analysis. *Methylospira* and *Methyloterricola*, which are the closest relatives of relative of *Ca.* Methylumidiphilus, were respectively in very low abundances or not detected in the samples, suggesting that 16S rRNA gene reads of Ca. Methylumidiphilus were not taxonomically assigned to its two closest genera. When we compared the abundance of *Ca*. Methylumidiphilus obtained with Kaiju to the abundances 16S rRNA sequences of unclassified ƴ-MO, the overall correlation was low (rho = 0.36), but this relation changed a lot depending on most the dominant ƴ-MO and the abundance of *Ca.* Methylumidiphilus. When the relative abundance of *Ca.* Methylumidiphilus was above 5 %, its correlation (rho) with the 16S rRNA gene-based abundances of uncultivated ƴ-MO raised to 0.75 (Supplementary Figure SM1C). This suggest that some of putative prokaryotic 16S rRNA sequences detected by SSU-ALIGN were very probably from *Ca*. Methylumidiphilus. This suggests that out hypothesis, that *Ca*. Methylumidiphilus might be a common MO that has escaped detection so far, is correct.

For α-MO the Spearman's rank correlation coefficient between the two 16S rRNA gene and Kaiju datasets was 0.69. This correlation value was strongly influenced by the samples where no α-MO were detected within the 16S rRNA sequences. If those 61 samples were removed from the data set, the Spearman's rank correlation coefficient was 0.78 (Supplementary Figure SM1D). As for MO and ƴ-MO, this indicates that both methods yield comparable results. Using the 16S rRNA gene approach, MOA and NC10-MO were not detected in most samples (152 and 115 respectively) and the correlations between the results of the two methods were poor (Supplementary figure SM1E and SM1F). Finally, no clear pattern was observed for V-MO (Supplementary figure SM1G).

The absence of low abundance MOs in many of the samples when the 16S rRNA gene method is used, suggests that this approach is more likely to miss low abundance taxa, due to the fact that the 16S rRNA gene presents only a fraction of the whole genome. Thus, whole genome based methods, such as Kaiju, detecting the whole genome are superior for the studying of the rare taxa. This is exemplified by the fact that for the 16S rRNA gene method only 1166193 reads were assigned to putative prokaryotic 16S rRNA sequences with most samples including less than 5500 reads that matched to the 16S rRNA gene. On the other hands the kaiju data set was composed of 2.56 * 10^8^ reads (1230562 reads per sample). In conclusion, the limited number of reads assigned to 16S rRNA gene combined with the absence of *Ca*. Methylumidiphilus strongly supports our approach of using Kaiju. Furthermore, we showed that for the taxa with abundances high enough to be detected by the 16S rRNA approach, both the protein-level classifier and the ribosomal RNA gene reads affiliation gives comparable results.


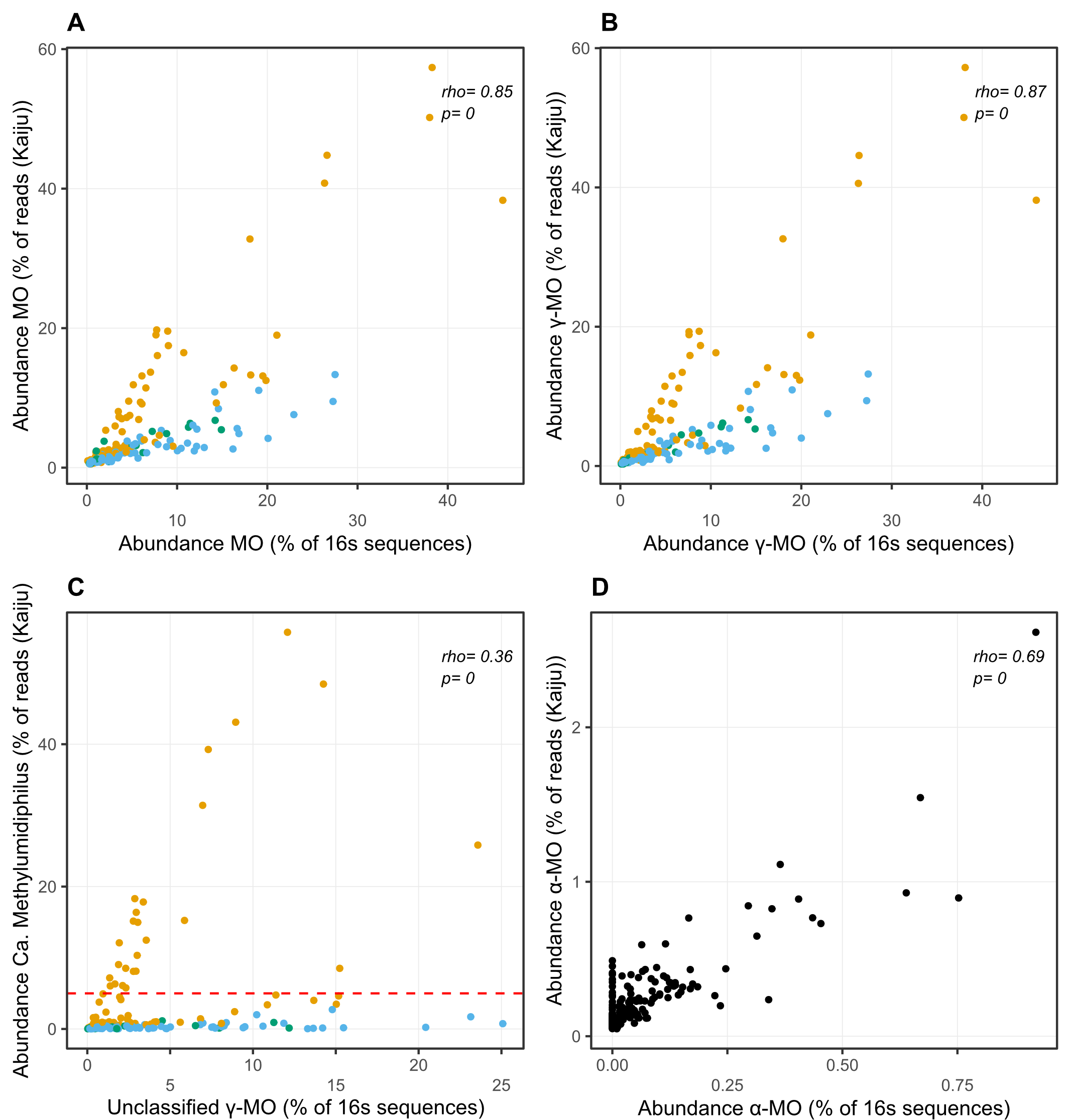


**
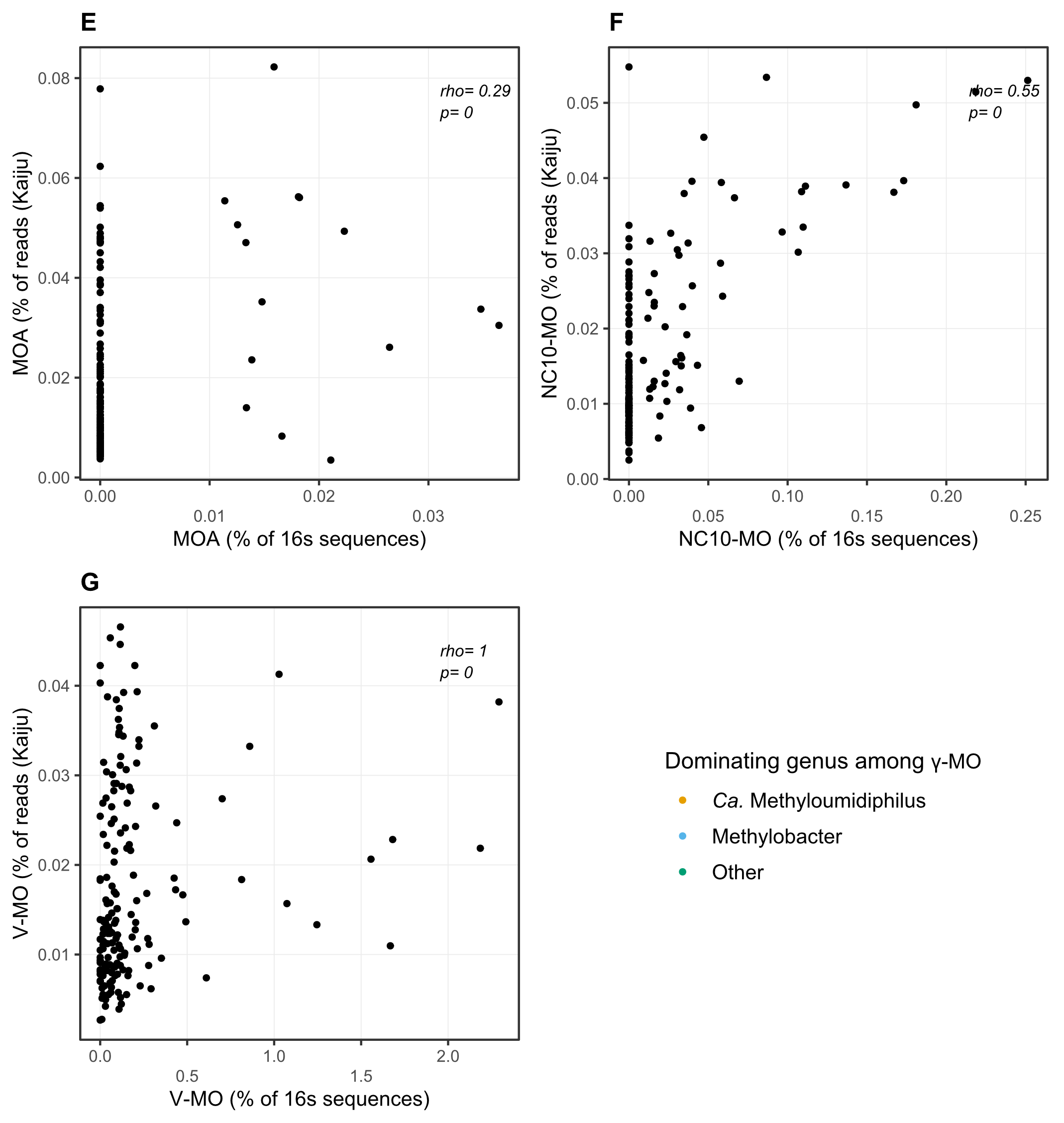
 Supplementary Figure SM1. Correlations between the relative abundances of MO taxa based on Kaiju and SSU-ALIGN (Putative prokaryotic 16S rRNA sequences) assignments.** Each panel (A-G) represent the correlation between relative abundances calculated for the same taxon but based on a different method of reads assignment. Abbreviations for the taxonomic groups are: alphaproteobacterial methanotrophs (α-MO), gammaproteobacterial methanotrophs (ƴ-MO), archaeal methanotrophs (MOA), bacterial phylum NC10 (NC10-MO), and Verrucomicrobial methanotrophs (V-MO). MO corresponds to the total methanotrophic community.

Color mapping was added in panels A, B and C to show which ƴ-MO genus dominated samples that included ƴ-MO. The dominating genera were determined using the Kaiju dataset. In panel C, the red dashed line marks a threshold at 5% of *Ca.* Methylumidiphilus. This threshold was used to calculate an approximate rho value for the set of samples dominated by *Ca.* Methylumidiphilus (the samples aligned on the left side of the plot). When only samples with *Ca.* Methylumidiphilus abundance above 5% are considered, the rho value is 0.75.
